# Switch to maraviroc with darunavir/r, both QD, in patients with suppressed HIV-1 was well tolerated but virologically inferior to standard antiretroviral therapy: 48-week results of a randomized trial

**DOI:** 10.1371/journal.pone.0187393

**Published:** 2017-11-21

**Authors:** Barbara Rossetti, Roberta Gagliardini, Genny Meini, Gaetana Sterrantino, Vincenzo Colangeli, Maria Carla Re, Alessandra Latini, Manuela Colafigli, Francesca Vignale, Stefano Rusconi, Valeria Micheli, Antonio Di Biagio, Giancarlo Orofino, Valeria Ghisetti, Alessandra Fantauzzi, Vincenzo Vullo, Pierfrancesco Grima, Daniela Francisci, Claudio Mastroianni, Andrea Antinori, Michele Trezzi, Lucia Lisi, Pierluigi Navarra, Benedetta Canovari, Antonella D’Arminio Monforte, Silvia Lamonica, Alessandro D’Avino, Maurizio Zazzi, Simona Di Giambenedetto, Andrea De Luca, for GUSTA trial study group

**Affiliations:** 1 Infectious Diseases Unit, Azienda Ospedaliera Universitaria Senese, Siena, Italy; 2 Clinic of Infectious Diseases, Catholic University of Sacred Heart, Rome, Italy; 3 Department of Medical Biotechnology, University of Siena, Siena, Italy; 4 Clinic of Infectious Diseases, Azienda Ospedaliera Universitaria Careggi, Firenze, Italy; 5 Clinic of Infectious Diseases, Azienda Ospedaliera Universitaria S.Orsola Malpighi, Bologna, Italy; 6 Microbiology, Azienda Ospedaliera Universitaria S.Orsola Malpighi, Bologna, Italy; 7 Infectious Dermatology and Allergology IRCCS IFO, Roma, Italy; 8 Clinic of Infectious Diseases, G. D’Annunzio University, Chieti, Italy; 9 Infectious and Tropical Diseases Unit, DIBIC L. Sacco Hospital, University of Milano, Milano, Italy; 10 Microbiology and Virology Laboratory, L. Sacco Hospital, Milano, Italy; 11 Infectious Diseases Unit, IRCCS S. Martino-IST, Genova, Italy; 12 Infectious Diseases Unit A, Amedeo di Savoia Hospital, Torino, Italy; 13 Microbiology and Virology Laboratory, Amedeo di Savoia Hospital, Torino, Italy; 14 Department of Clinical Medicine, Sapienza University of Rome, Roma, Italy; 15 Department of Public Health and Infectious Diseases, Sapienza University of Rome, Roma, Italy; 16 Division of Infectious Diseases, S. Caterina Novella Hospital, Galatina, Lecce, Italy; 17 Clinic of Infectious Diseases, University of Perugia, Perugia, Italy; 18 Infectious Disease Unit, SM Goretti Hospital, Department of Public Health and Infectious Diseases, Sapienza University, Latina, Italy; 19 Infectious Diseases Unit, IRCCS L. Spallanzani, Roma, Italy; 20 Infectious Diseases Unit, Pistoia Hospital, Pistoia, Italy; 21 Pharmacology Department, Catholic University of Sacred Heart, Rome, Italy; 22 Infectious Diseases Unit, Pesaro Hospital, Pesaro, Italy; 23 Infectious and Tropical Diseases Institute, Department of Health Sciences, University of Milan San Paolo Hospital, Milan, Italy; University of Ottawa, CANADA

## Abstract

**Objectives:**

Primary study outcome was absence of treatment failure (virological failure, VF, or treatment interruption) per protocol at week 48.

**Methods:**

Patients on 3-drug ART with stable HIV-1 RNA <50 copies/mL and CCR5-tropic virus were randomized 1:1 to maraviroc with darunavir/ritonavir qd (study arm) or continue current ART (continuation arm).

**Results:**

In June 2015, 115 patients were evaluable for the primary outcome (56 study, 59 continuation arm). The study was discontinued due to excess of VF in the study arm (7 cases, 12.5%, vs 0 in the continuation arm, p = 0.005). The proportion free of treatment failure was 73.2% in the study and 59.3% in the continuation arm. Two participants in the study and 10 in the continuation arm discontinued therapy due to adverse events (p = 0.030). At VF, no emergent drug resistance was detected. Co-receptor tropism switched to non-R5 in one patient. Patients with VF reported lower adherence and had lower plasma drug levels. Femoral bone mineral density was significantly improved in the study arm.

**Conclusion:**

Switching to maraviroc with darunavir/ritonavir qd in virologically suppressed patients was associated with improved tolerability but was virologically inferior to 3-drug therapy.

## Introduction

Antiretroviral therapy (ART) significantly reduces morbidity and mortality associated with HIV infection; this is best achieved by chronically maintaining undetectable levels of HIV-1 RNA in patients plasma [1]. The combination of three active agents in the first-line regimen is the standard of care to achieve this virological goal and nucleoside reverse transcriptase inhibitors (NRTIs) are the essential component of all initial treatment strategies [2–4].

The long-term side effects of NRTIs are still of concern. Some agents are associated with renal and bone toxicity [5–7], others with increased rates of cardiovascular events [8]. Therefore, several attempts have been performed in order to test NRTI-sparing maintenance therapy in virologically controlled individuals [9–12]. Maraviroc is the only CCR5 (R5) co-receptor antagonist approved for use in patients carrying an R5-tropic virus [13]. Currently, the drug is approved only for patients failing previous regimens, given its inferior efficacy as initial therapy [14–16]. Nonetheless, agents with this mechanism of action should ideally be used early during treatment, before the possible switch from R5 to non-R5 tropism at later stages [17].

The efficacy of maraviroc as maintenance therapy in virologically controlled patients has been recently tested in the MARCH study [18]. In this large randomized study enrolling virologically controlled patients on 2 NRTIs plus a boosted protease inhibitor (bPI), substitution of NRTIs with maraviroc twice daily was inferior to continuing the baseline 3-drug regimen. One obstacle to the implementation of this strategy, the requirement of viral tropism testing in patients with suppressed viremia, has been overcome by genotypic tropism testing on viral DNA [19]. This strategy has now been validated retrospectively [20–21] as well as prospectively [18]. The MARCH study was limited as the switch to maraviroc included patients with boosted PI from older generations that were in most cases administered twice daily. Also, maraviroc was used twice daily, which lacks convenience in terms of dosing and cost.

The aim of this study was to test non-inferiority of an NRTI-sparing dual therapy based on once daily maraviroc with darunavir/ritonavir as compared to continuing an ongoing, virologically successful 3-drug therapy. The experimental regimen tested here is not according to the label of maraviroc. A secondary aim was to perform a prospective validation of selecting patients with R5 virus based on single-sequence viral DNA genotyping [22].

## Methods

### Study design, inclusion and exclusion criteria

GUSTA (GUided Simplification with Tropism Assay) was a multicenter, open-label, randomized study (registered at www.clinicaltrials.gov, number NCT01367210). The study protocol was authorized by the Italian Drug Regulatory Agency (AIFA) and approved by the local Ethics Committees at each study center; written informed consent was obtained from all patients before participation. The study was performed in accordance with the ethical guidelines of the Declaration of Helsinki (7th revision) and with the International Conference on Harmonization Good Clinical Practice guidelines. The authors confirm that all related trials for this intervention are registered. Study approval by the Ethics Committee of the Catholic University was obtained on November 18, 2010. Between May 23, 2011 and April 22, 2015, patients underwent screening for the study and the latest visit was on September 28, 2015. The complete list of Ethics Committes that approved study is: Ethics Committee of Umbria and Perugia; Ethics Committee of Azienda Ospedaliera Sacco, Milan; Ethics Committee of Modena; Ethics Committee Azienda Ospedaliero-Universitaria of Florence; Ethics Committee University of Study D’Annunzio and Chieti; Ethics Committee AUSL 9 of Grosseto; Ethics Committee Azienda Ospedaliera Universitaria S Martino, Genova; Ethics Committee AUSL 3 Pistoia; Ethics Committee Azienda Ospedaliera S. Salvatore of Pesaro; Ethics Committee AOU San Luigi Gonzaga of Orbassano; Ethics Committee Azienda Ospedaliero-Universitaria Policlinico S. Orsola-Malpighi of Bologna; Ethics Committee IRCC of Rome; Ethics Committee of Lecce; Ethics Committee Policlinico Umberto I of Rome; Ethics Committee Lazio 2; Ethics Committee IRCC Spallanzani of Rome; Ethics Committee San Paolo Hospital, Milano.

Eligible patients were HIV-1-infected, aged ≥18 years, treated with any ART regimen including 3 drugs without treatment changes during the last 3 months, with HIV-1 RNA <50 copies/mL for at least 6 months on two consecutive determinations, with CD4 cell count >200 cells/mm^3^ for at least 3 months, and with R5 virus.

Main exclusion criteria were having a CD4 cell count at nadir <50 cell/mm^3^ or <100 cell/mm^3^ in case of a history of virological failure with PI, past use of maraviroc, pregnancy, breastfeeding status, positive serum hepatitis B virus surface antigen, a history of major toxicity to any of the study or current drugs, AIDS-related events in the year before screening, liver cirrhosis, eGFR <30 mL/min and grade IV ALT or AST elevation. Previous virological failures were allowed, but previous failures to enfuvirtide or integrase inhibitors and patients with at least one major or two minor darunavir resistance mutations (last available IAS-USA list) were excluded.

### Determination of viral tropism and resistance testing

All patients underwent genotypic testing for HIV-1 coreceptor tropism at screening by population sequencing on whole blood using a single DNA sequence of the gp120 V3 env region [22]. DNA sequence retesting was allowed a maximum of three times in case of amplification failure. Results were interpreted using the geno2pheno prediction algorithm. A false positive rate (FPR) higher than 10% indicated an R5 virus. The genotyping was performed at virological laboratories that had been certified by a national QC/QA program for genotypic tropism determination [23]. The genotypic HIV-1 coreceptor tropism assay has been deposited in the protocols.io repository with doi 10.17504/protocols.io.jmnck5e. Genotypic resistance to NRTIs, NNRTIs and PIs was determined using the Viroseq HIV-1 Genotyping System.

### Study procedures

At baseline, eligible patients were randomized 1:1 to receive maraviroc 300 mg qd (except those with glomerular filtration rate, eGFR, <80 mL/min who received 150 mg qd) plus darunavir/ritonavir 800/100 mg (study, S, arm) or to continue the previous antiretroviral regimen (continuation, C, arm). Follow-up visits were scheduled at weeks 4, 12 and every 12 weeks thereafter. Routine physical examination and laboratory tests, including CD4 cell counts and HIV-1 RNA levels, were performed at baseline and at each follow-up visit.

### Efficacy and safety outcomes definition and handling

Treatment failure was defined as any of the following: virological failure, discontinuation or change of any current drug due to any cause, withdrawal of consent after treatment initiation, loss to follow-up, progression to AIDS or death. Virological failure was defined as the second of two consecutive HIV-1 RNA levels >50 copies/mL or a single HIV-1 RNA level >1,000 copies/mL. At the time of virological failure, an additional visit and blood sampling were performed. Clinical and laboratory adverse events occurring during the study were graded according to the latest available Division of AIDS tables. A Data Safety Monitoring Board was established to monitor patient safety and treatment efficacy data.

### Study endpoints and sample size calculation

The primary study endpoint was the proportion of patients free of treatment failure at 48 weeks on the per-protocol population. Based on the assumption that 90% of patients in the continuation arm would meet the primary outcome, enrolment of 330 patients distributed 1:1 in each study arm was planned in order to test non-inferiority of the study arm with a lower bound of 10%, a confidence interval of 95% and a power of 80%. Secondary endpoints were: the proportion of patients with treatment failure and virological failure at 48 weeks on the intention-to-treat population, modification of CD4 cell count and of blood metabolic parameters, changes in bone parameters, self-reported adherence and symptoms scores, and of health-related Quality of Life from baseline to week 48.

### Evaluation of bone mineral density

At baseline and at week 48, patients from 7 study sites underwent dual-energy X-ray absorptiometry scans to analyze bone mineral density (BMD) (Hologic Inc., Waltham, MA, USA). Total L2–L4 lumbar column and femoral neck BMD were recorded. Moreover, plasma alkaline phosphatase (total and bone-associated), 25(OH)vitamin D, parathyroid hormone (PTH) and osteocalcin were measured.

### Plasma drug levels, adherence, patient-reported symptoms and quality of life

Plasma drug levels were measured in arm S in blood collected before morning antiretroviral drug intake between week 4 and 48 by a validated tandem mass spectrometry (UPLC-MS/MS). The time of the last drug intake and of blood sampling were recorded for each patient and C_trough_ were calculated accordingly using the formula C = C_0_ × e^-kt^ (C = calculated trough levels; C_0_ = at the time measured; K = elimination rate consant (0.693/t_1/2_); e = base of natural logaritm). Liquid/liquid extraction was employed for purification and concentration of maraviroc, darunavir and ritonavir from plasma samples. Samples were injected into an AQUITY UPLC system employing an AQUITY UPLC BEH C18 column (Waters Corp., Milford, MA, USA) for analysis. The UPLC was connected to a triple quadrupole tandem mass detector (Waters Corp., Milford, MA, USA) for the mass spectrometric detection. Data were processed using MassLynx with a QuanLynx program version 4.1 (Waters Corp., Milford, MA, USA). The detection limit for the drug determination was 30 ng/mL. We computed C_trough_ when maraviroc levels were available 6–27 hours after intake and darunavir 10–27 hours after intake, i.e. during the linear phase of the elimination curve. All patients were instructed to take the study medications with food. In both arms, self-reported adherence (weeks 0, 4, 24 and 48), patient-reported symptoms and physical/mental Quality of Life (QoL) scores (weeks 0 and 48) were collected using validated questionnaires [24, 25]. Self-reported adherence was recorded as indicated on a 0–100 visual analogue scale (VAS). Both physical and mental health-related QoL were assessed on a 0–100 VAS scale (0 = worst level; 100 = best level). In addition, the questionnaire included a list of 32 common self-reported symptoms, which were graded between 0 (absent) and 5 (very intense): mean values of symptoms were compared between study arms.

### Statistical analysis

Descriptive statistics were used to describe baseline patients characteristics, main study outcomes, laboratory toxicities, clinical adverse events and serious adverse events. Differences between categorical variables were tested by the Chi-square test, differences between continuous variables were tested using the t-test or the Mann-Whitney U test, as appropriate. Changes from baseline values at week 48 were assessed using Student’s t-test for paired samples. The primary efficacy endpoint, treatment failure, was investigated by per-protocol (PP) analysis at study week 48: this analysis excluded patients with major protocol violations. In addition, an intention-to-treat (ITT) analysis at study week 48 including all randomized patients who received at least one drug dose after randomization was also performed. In all the mentioned analyses on treatment failure, missing values and those who had changed or discontinued their ART regimen were counted as failures. In a further secondary analysis, time to virological failure was also analysed in all randomized patients divided by study arm, until last available follow-up at the time of study interruption, using the Kaplan-Meier method. Non-inferiority for the primary outcome was tested using the one-sided test procedure: noninferiority would be established at an alpha level of 0.05 if the dual therapy arm achieved the primary outcome in no more than 10% less patients than the current therapy arm.

A two tailed P value <0.05 was considered to be statistically significant. All analyses were performed using the SPSS version 22 software package (IBM, SPSS, Chicago, IL, USA, IBM).

## Results

### Patient characteristics at baseline

**Fig 1:**
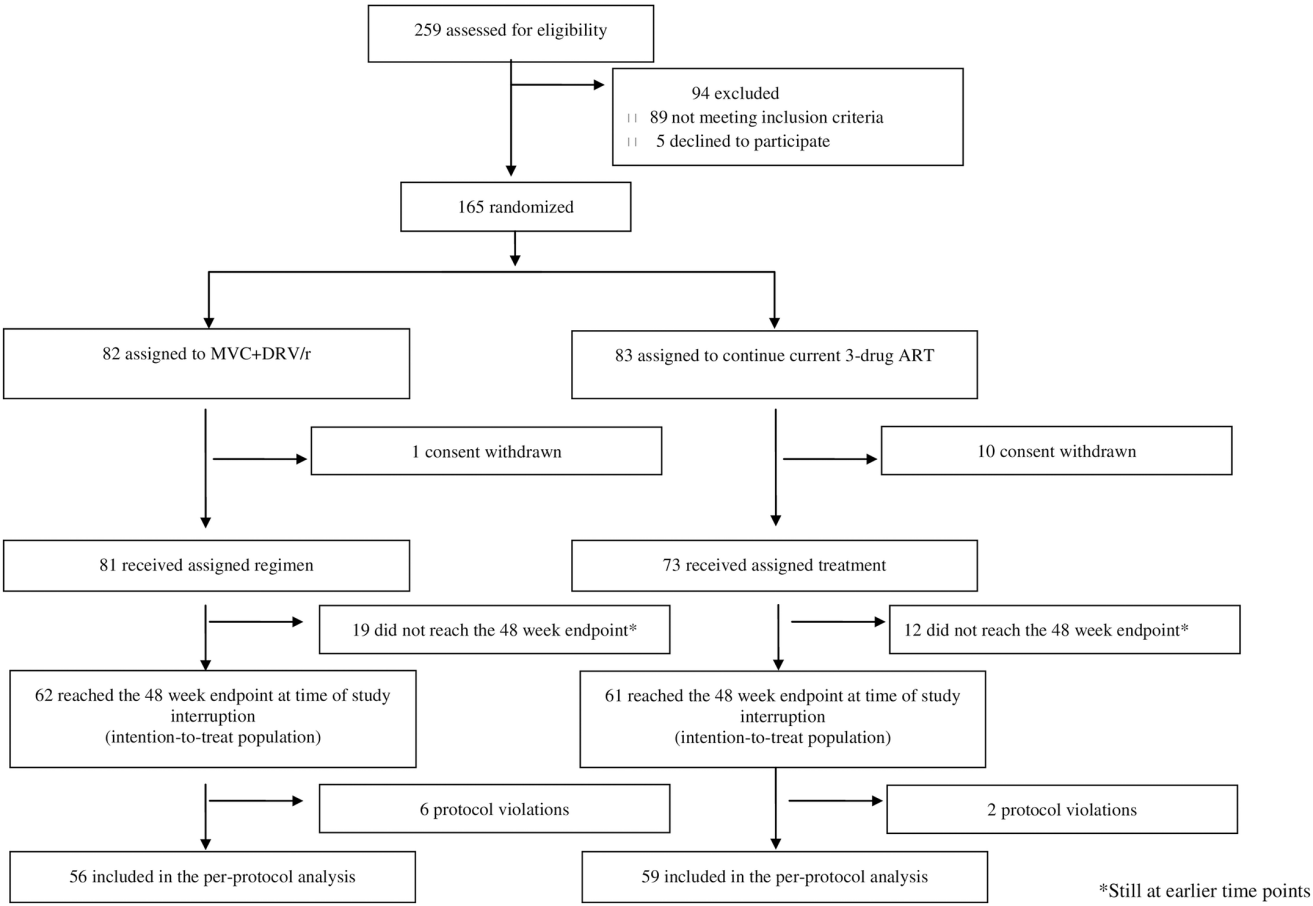
GUSTA flow diagram.

Between May 23, 2011 and April 22, 2015, 259 patients underwent screening for the study. On June 2015, based on a pre-planned 48-week interim analysis, the Data Safety Monitoring Board recommended trial discontinuation due to an excess of virological failures in the study arm. At that time, screening failures were 89/259 (36%): 67 (26%) for non-R5 viral tropism, 11 (4%) for HIV-RNA >50 copies/mL, 6 (2%) for V3 region of HIV-1 gp120 not amplifiable, 5 (2%) due to other exclusion criteria. Five (2%) additional patients were excluded due to consent withdrawal before randomization. Therefore, 165 patients were randomized of whom 123 were evaluable for the 48-weeks ITT analysis (62 in arm S and 61 in arm C), and 115 were included in the main PP analysis (56 in arm S and 59 in arm C) (Fig 1).

The main baseline characteristics of the PP study population, overall and divided by study arm, are summarized in Table 1, while the respective details for the ITT population are summarized in S1 Table.

**Table 1:** Baseline patients characteristics of the per-protocol population.

	Total n = 115	DRV/r + MVC (Arm S) n = 56	3-drug ART (Arm C) n = 59	P-value (between arms)
Age, years*	49 (41–57)	51 (44–59)	48 (40–55)	0.109
Male gender	86 (75)	43 (77)	43 (73)	0.630
Caucasian ethnicity	104 (90)	50 (89)	54 (91)	0.280
Risk factor:				0.223
Heterosexual	46 (40)	24 (43)	22 (37)	
Homo/bisexual	40 (35)	20 (36)	20 (34)	
Past injecting drug user	11 (9)	7 (12)	4 (7)	
Other/unknown	18 (16)	5 (9)	13 (22)	
HCV co-infection	16 (14)	9 (16)	7 (12)	0.322
Years from HIV diagnosis*	12 (7–18)	14 (7–19)	11 (7–17)	0.237
Years from first ART initiation*	10 (6–16)	10 (6–16)	10 (6–15)	0.610
Months from last regimen initiation*	51 (34–69)	51 (36–70)	50 (29–64)	0.324
Months from last HIV-RNA >50 cp/mL**	59 (52–65)	59 (51–68)	58 (49–67)	0.824
CD4 nadir, cells/μL*	222 (137–310)	214 (108–314)	222 (139–304)	0.765
CD4, cells/μL*	659 (495–923)	640 (494–992)	690 (494–908)	0.688
Treatment at screening:				
NRTI	109 (95)	51 (91)	58 (98)	0.081
TDF	70 (61)	31 (55)	39 (66)	0.238
NNRTI	22 (19)	9 (16)	13 (22)	0.416
InSTI	22 (19)	11 (17.7)	11 (19)	0.892
PI	68 (59)	36 (64)	32 (54)	0.273
Boosted PI	57 (50)	30 (54)	27 (46)	0.403
DRV/r	30 (26)	14 (25)	16 (27)	0.796
QD regimen at screening	72 (63)	34 (61)	38 (64)	0.074

Results are expressed as n (%),

*median (IQR) or

** mean (95% CI).

Abbreviations legend: DRV/r, darunavir/ritonavir; MVC, maraviroc; ART, antiretroviral therapy; TDF/FTC, tenofovir/emtricitabine; HCV, hepatitis C virus; PI, protease inhibitor; NNRTI, non-nucleoside reverse transcriptase inhibitor; NRTI, nucleoside reverse transcriptase inhibitor; InSTI, Integrase strand transfer inhibitors; QD, once daily

### Main study outcomes: Treatment and virological failure

**Fig 2:**
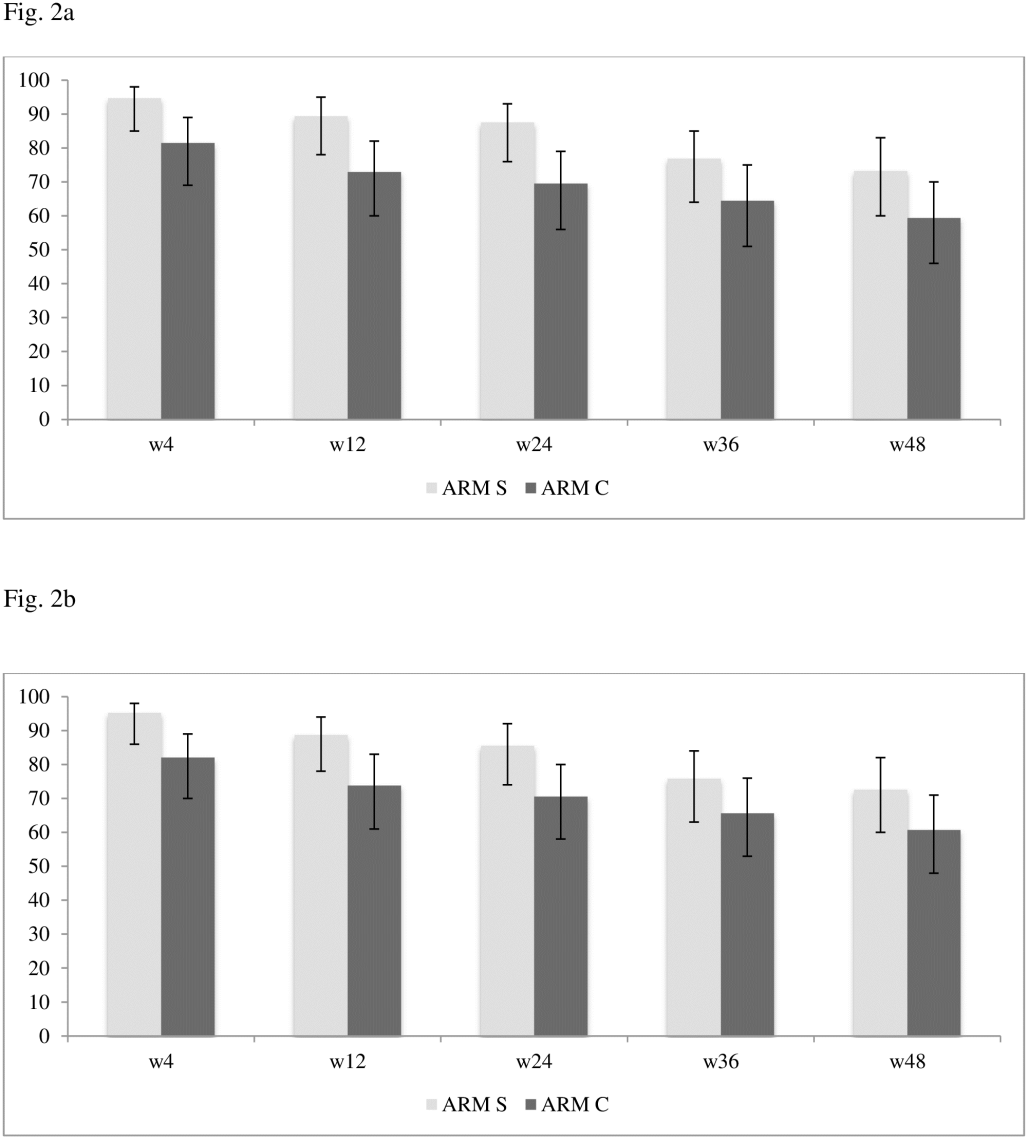
Proportion of individuals without treatment failure over the 48 study weeks by randomization arm in the (a) per protocol population and (b) intention to treat population. Arm S = study arm (switch to maraviroc + darunavir/ritonavir); Arm C = continuation arm (continuation of previous 3-drug therapy).

In the PP population, 76/115 (66%) patients were free of treatment failure at 48 weeks: 41/56 (73.2%) in arm S and 35/59 (59.3%) arm C (Fig 2a). The proportion free of treatment failure in arm S was noninferior to the proportion in arm C (S minus C arm +13.9%; 95%, CI -0.6; +28.4, p = 0.058). Detailed reasons of treatment failures are shown in Table 2. Seven protocol-defined virological failures occurred in arm S (12.5%) and none in arm C (p = 0.005). Two patients (3.6%) in arm S and 10 (16.9%) in arm C discontinued therapy owing to adverse events (p = 0.030).

**Table 2:** Causes of treatment failure.

**Per protocol population**			
	**DRV/r + MVC (Arm S) n = 56**	**3-drug cART (Arm C) n = 59**	**p-value**
Any cause	16 (28.6)	24 (40.7)	0.238
Virological Failure	7 (12.5)	0 (0)	0.005
Adverse events (potentially treatment-related)	2* (3.6)	8** (14.3)	0.095
Adverse events (all)	2 (3.6)	10 (16.9)	0.030
Withdrawal of consent/Patient’s choice	2 (3.6)	9 (16)	0.054
Loss to follow up	3 (5.4)	3 (5)	1.000
Other	2 (3.6)	2 (3.4)	1.000
**Intention-to-treat population**			
	**DRV/r+ MVC (Arm S) N = 62**	**3-drug cART (Arm C) N = 61**	**p**
Any cause	17 (27.4)	24 (39.3)	0.226
Virological Failure	8 (12.9)	0 (0)	0.006
Adverse events (potentially treatment-related)	2* (3.2)	8** (13.1)	0.054
Adverse events (all)	2 (0)	10 (16.4)	0.016
Withdrawal of consent/Patient’s choice	2 (3.2)	9 (14.8)	0.030
Loss to follow up	3 (4.8)	3 (4.9)	1.000
Other	3 (4.8)	2 (3.3)	1.000

*1 Asthenia, 1 rash,

** 5 Osteoporosis/osteopenia, 2 creatinine increase, 1 diarrhea

In the ITT population the proportion free of treatment failure at 48 weeks in arm S was noninferior to the proportion in arm C (S arm minus C arm +11.9%; 95%CI -2.1; +25.9, p = 0.080) (Fig 2b). All 8 protocol-defined virological failures in the ITT population occurred in arm S. Virological blips without protocol-defined virological failure occurred in seven patients in arm S, and four in arm C. In 7 of 7 successfully genotyped patients no resistance-associated mutation was detected in the reverse transciptase and protease region, viral tropism remained R5 in 6 of 6 succesfully genotyped viral RNA samples and in 7 of 8 DNA samples (1 patient harbored a non-R5 virus with a FPR of 0.5%, RNA genotyping of this case was repeatedly unsuccessful). Four to 8 weeks after virological failure HIV-1 RNA was <50 copies/mL without any treatment change in one patient and after switching to a triple therapy in the remaining seven.

**Fig 3:**
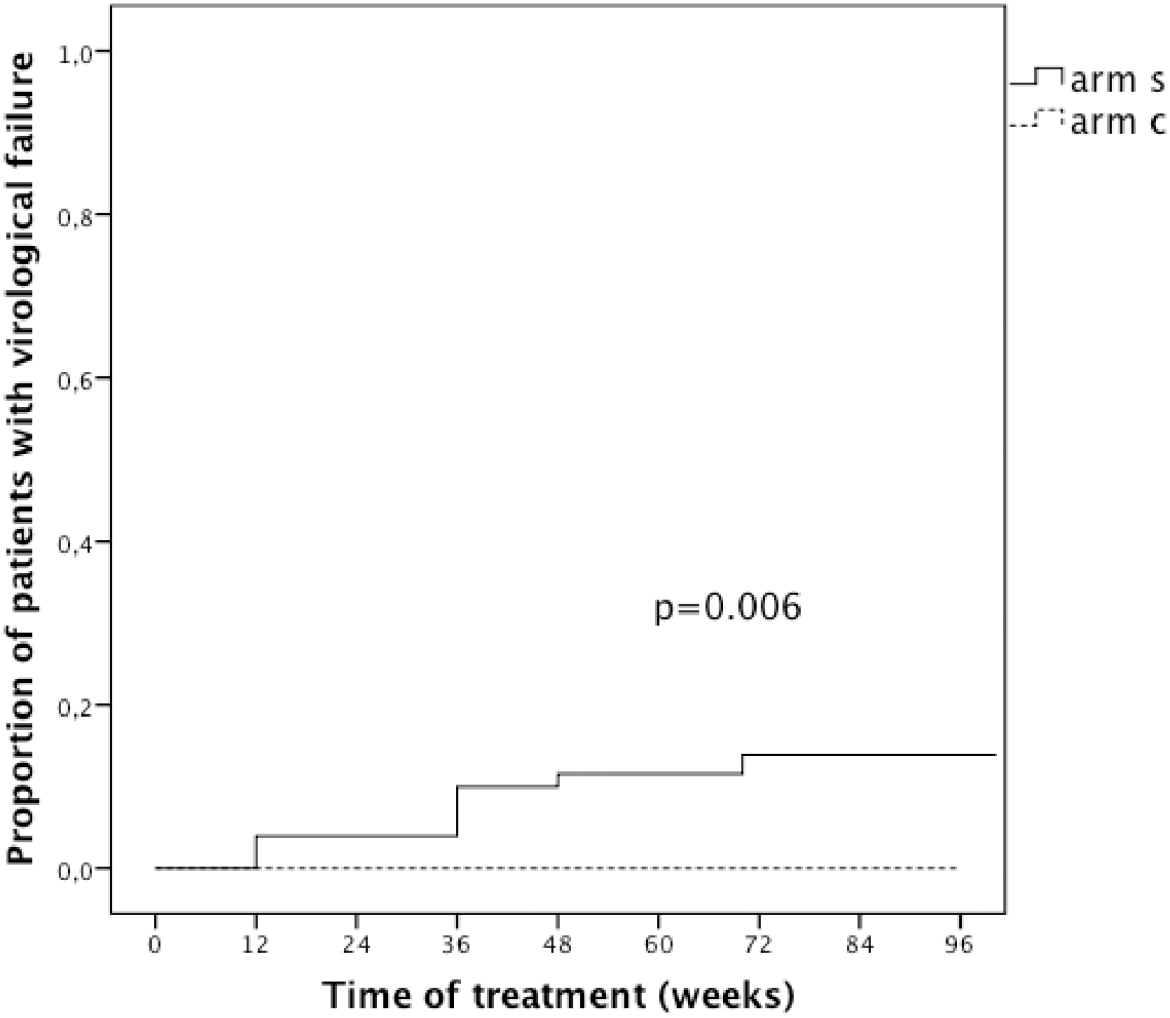
Proportion of individuals with virological failure over the complete follow-up available until the time of study interruption in the intention to treat population. Arm S = study arm (switch to maraviroc + darunavir/ritonavir); Arm C = continuation arm (continuation of previous 3-drug therapy).

When baseline characteristics of the 8 patients with virological failure in arm S (ITT population) were compared with the 54 without virological failure in the same arm, significant differences included a higher mean baseline FPR of the geno2pheno co-receptor assay (67% vs 42%, p = 0.015), a shorter duration of previous virological suppression (37 vs 61 months, p = 0.057) in those with virological failure as compared to those without. Other characteristics (CD4 cell count at nadir and at baseline, time from HIV diagnosis or from first cART initiation, cART drugs at screening and HCV status) did not differ between patients with virological failure and those without (S2 Table). In a further analysis we described the time to virological failure using the complete follow-up available until the time of study interruption. While there were no failures in arm C, the mean time to virological failure in arm S was 30 weeks (95% CI 19–42) (Fig 3).

### Clinical and laboratory events

During the 48 weeks of the study 78 clinical adverse events were observed in 49 of the 123 patients (ITT population, S3 Table). Eleven were classified as serious adverse events: 6 in 5 patients in arm S (1 uterine leiomyoma, 1 sub-ileus, 1 traumatic bone fracture, 1 urinary tract infection, 1 syncope, 1 fibromyalgia) and 5 events in 4 patients in arm C (1 death for myocardial infarction, 1 pericarditis, 1 traumatic bone fracture, 1 fever/chest pain, 1 prostatic adenocarcinoma). None of the serious adverse events was considered to be drug-related. Clinical adverse events of grade 1 or 2 were 37 in 22 patients in arm S and 30 in 23 patients in arm C (p = 0.27). No grade 3 clinical adverse event occurrred. Two events determined study discontinuation at week 4 in arm S (asthenia and rash, both grade 1) and 10 in arm C (4 osteoporosis at baseline, w4, w27, w48, 2 renal function deterioration at w4 and w12, diarrhoea at w24, insomnia at w12, death for miocardial infarction at w12 and prostatic adenocarcinoma at w36.

Twenty-eight (45%) and 14 (23%) patients in arm S and 24 (41%) and 19 (31%) in the arm C experienced grade 3 and grade 4 laboratory toxicities, respectively, without significant differences between arms.

### Changes in cd4 cell count, blood lipids and renal function

**Fig 4:**
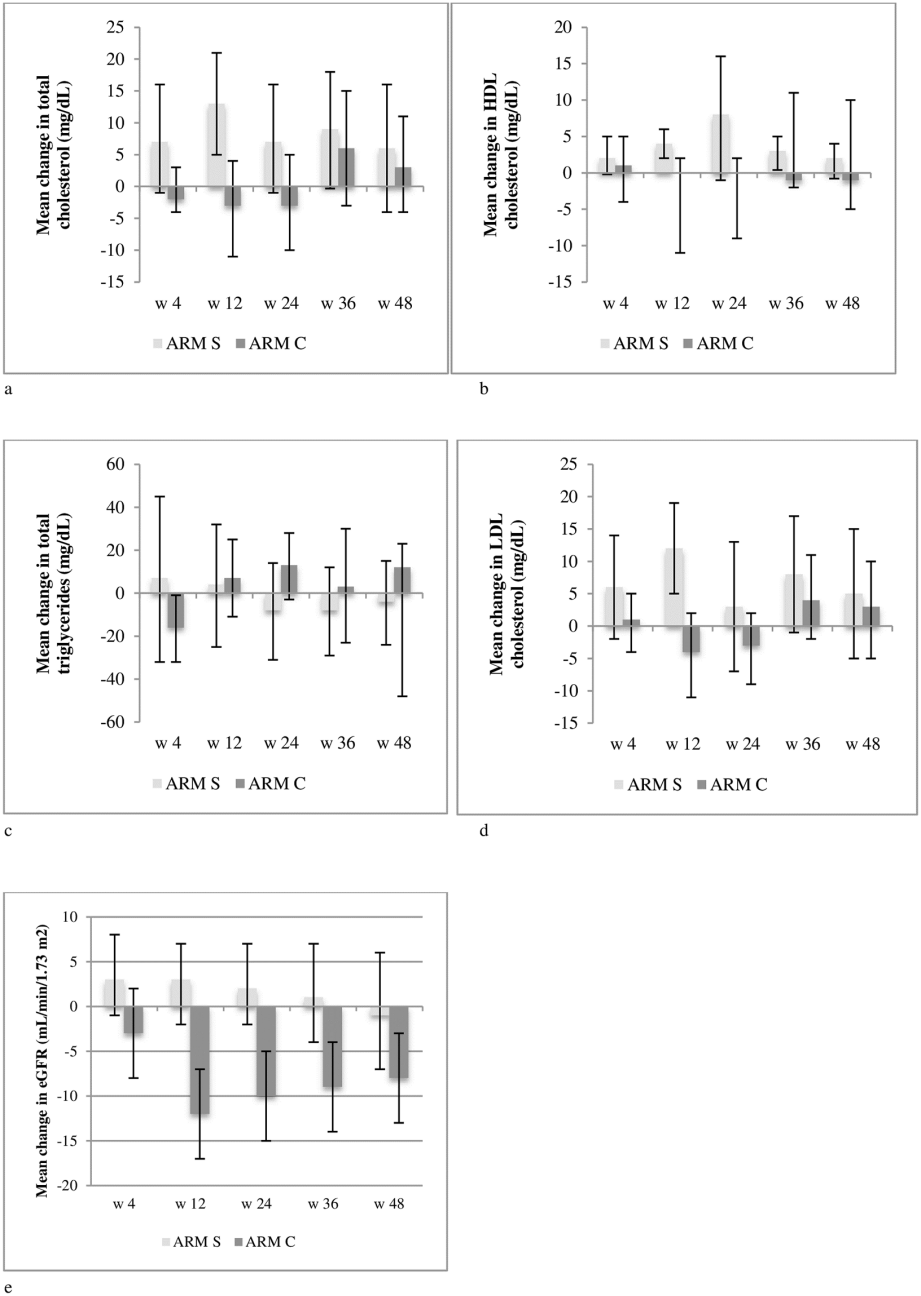
Evolution of (a-d) blood lipids and (e) renal function as by estimated GFR (CKD-EPI) over the 48 study weeks by randomization arm. Arm S = study arm (switch to maraviroc + darunavir/ritonavir); Arm C = continuation arm (continuation of previous 3-drug therapy).

Changes in CD4 cell counts did not differ between the two study arms. At week 48, triglycerides, total cholesterol, low-density lipoprotein (LDL) and high-density lipoprotein (HDL) cholesterol did not show significant modifications (Fig 4a–4d). There were no differences in changes from baseline eGFR between the 2 study arms at week 48 (mean -1 mL/min/1.73m^2^ in arm S, vs. -8 mL/min/1.73m^2^ in arm C, p = 0.12), although the study arm showed a better preservation of kidney function from baseline between week 12 and 36 (at week 12 and 24 p = 0.004, at week 36 p = 0.034) (Fig 4e).

### Bone parameters

Changes in BMD and bone metabolism markers were evaluated in 64 patients: 34 in the S arm and 30 in the C arm. At 48 weeks, patients in arm S showed significantly better gains over those in arm C in femoral neck BMD (mean change +1.38% [SD 4.45%] in arm S vs -2.35% [8.32%] in arm C, p = 0.04). Lumbar column BMD changes did not show significant differences between arms (at 48 weeks mean change from baseline +0.54% [8.09%] in arm S vs -0.74% [6.32%] in arm C, p = 0.52). Patients in arm S showed a more prominent reduction of total plasma alkaline phosphatase (-15.54 U/L [10.82 U/L] vs -1.45 U/L [13.36 U/L] in arm C, p<0.001) and bone alkaline phosphatase (-9.10 μg/L [0.33 μg/L] vs -1.45 μg/L [4.69 μg/L] in arm C, p = 0.19) at 48 weeks, compatible with a reduced bone remodeling. No other significant changes were observed in the other bone metabolism biomarkers.

### Drug levels, adherence, patient-reported symptoms and health-related quality of life

One-hundred fourteen patients of the ITT population (62 in arm S, 52 in arm C) had at least one assessment of self-reported adherence, patient-reported symptoms and QoL: baseline characteristics of these patients did not differ from those of the main stydy population (not shown). Detailed numbers are reported in Table 3. Drug levels were measured in the 62 patients in arm S. In 8 patients C_trough_ of all drugs at virological failure were significantly lower as compared to those of patients without virological failure: darunavir median level 681 ng/mL (IQR 0–1,589) vs 1,368 ng/mL (1,048–1,646), p = 0.038, maraviroc 36 ng/mL (7–152) vs 121 ng/mL (100–127), p<0.001, and ritonavir 2 ng/mL (0–81) vs 33 ng/mL (17–49), p<0.001. Adherence, patient-reported symptoms, physical health or mental health-related QoL scores did not differ between arms at baseline and at week 48 (Table 3). Physical health-related QoL declined significantly from baseline to 48 weeks in arm C (63.64% vs 53.64%, p = 0.004) but not in arm S (65.18% vs 58.33%, p = 0.26) (Table 3). Mean adherence was significantly lower in patients during virological failure (arm S) and treatment failure (arm C) (Table 3). Among patients with self-reported adherence ≤80% in at least one determination, 5 of 30 (16.7%) in arm S versus 0 of 21 (0%) in arm C showed virological failure (p = 0.049).

**Table 3:** Adherence, health-related QoL and patient-reported symptoms at different time points, based on randomization arm.

	DRV/rit + MVC (Arm S)			3-drug cART (Arm C)			Between arm comparisons	
	**Baseline (n = 34)**	**Week 48 (n = 34)**	**P**^	**Baseline (n = 27)**	**Week 48 (n = 27)**	**P**^	**P (baseline)**	**P (week 48)**
Self reported adherence (VAS 0–100%)	87.35% (16.57)	87.35% (11.36)	1.00	88.88% (13.95)	89.63% (14.00)	0.80	0.70	0.48
	**Baseline (n = 30)**	**Week 48 (n = 22)**		**Baseline (n = 30)**	**Week 48 (n = 22)**			
Patient-reported symptoms score	1.39 (0.31)	1.44 (0.38)	0.21	1.41 (0.31)	1.36 (0.27)	0.35	0.80	0.42
Mental health QoL (VAS 0–100%)	62.07% (25.54)	63.33% (22.48)	0.55	57.95% (22.34)	56.82% (25.79)	0.83	0.55	0.33
Physical health QoL (VAS 0–100%)	65.18% (20.79)	58.33% (20.05)	0.26	63.64% (16.67)	53.64% (26.60)	0.04	0.78	0.47
	At failure (n = 8)*	During success*** **(n = 129)**	**P**	At failure (n = 12)**	During success*** **(n = 108)**	**P**		
Self reported adherence (VAS 0–100%)	73.3% (20)	88.2% (12.5)	0.001	80.8% (17.8)	89.23% (13.7)	0.05		

Values indicate means (standard deviations). The symptoms score reports the sum of the values of the intensity of the symptom (from 1 = absent to 5 = very much) divided by the number of evaluable symptoms per patient (max 30 total symptoms): adapted from ISS QoL [25].

^P-values of within-arm comparisons (week 48 vs baseline; success vs failure).

* In arm S all failures were virological;

** in arm C all failures were non-virological;

***instances with contemporary HIV RNA <50 copies/mL and no other cause of failure.

## Discussion

This open-label randomized trial in virologically controlled patients on a 3-drug ART with R5 virus was designed to demonstrate the non-inferiority of switching therapy to once daily maraviroc plus ritonavir-boosted darunavir as compared to continuing the previous 3-drug regimen. Although the coadministration of once daily maraviroc 300 mg with darunavir/ritonavir 800/100 mg is not according to label, other studies demonstrated high tolerability and favourable pharmacokinetics when compared with 300 mg of maraviroc twice daily with 245 mg of tenofovir/200 mg of emtricitabine [26]. The study was prematurely interrupted due to an excess of virological failures in the dual therapy arm at week 48. The main study outcome, the proportion without treatment failure at 48 weeks, did not differ between arms. Similarly, in a population of virologically suppressed patients, the MARCH study showed inferior efficacy of maraviroc administered twice daily with one of several different bPIs as compared to continuing 2 NRTIs plus bPI [18]. The present study and MARCH differ in patients treatment history, PI types used, maraviroc dosing and virological outcomes definition, but both studies indicate that maraviroc with bPI is inferior to standard 3-drug therapies in treatment experienced, virologically suppressed patients. Both studies also complement results from the MODERN trial showing inferior efficacy of maraviroc 150 mg qd as compared to tenofovir/emtricitabine, both with darunavir/ritonavir in treatment naïve patients [15]. In order to explain the reason for the excess of virological failures of this dual therapy regimen we examined a number of factors. At virological failure no case developed drug resistance and we found a single case of tropism switch detected in viral DNA. Patients with virological failure had significantly lower drug levels and a lower self-reported adherence. In addition, patients with 80% or lower adherence in the S arm had 17% risk of virological failure, while no failures occurred in patients with such low adherence levels in the C arm. Altogether this suggests a lower “forgiveness” of the maraviroc plus darunavir/ritonavir regimen as compared to 3-drug ART, so that during periods of suboptimal adherence, the insufficient drug exposure does not maintain virological suppression.

For selecting candidate patients with R5-tropic virus we used a lower cut-off of the FPR of the geno2pheno interpretation system (10%) than suggested by a European panel (20%) [19]. Notably, we found no correlation between this less stringent criterion and virological failure. Indeed, virological failure was not associated with lower geno2pheno FPR at the screening assay. All but one case of virological failure had FPR >60% at screening, which is associated with very low rates of X4 or dual/mixed viral quasispecies [27], and the average FPR was actually higher in those failing than in those subsequently maintaining viral suppression. Therefore, the choice of a single genotypic tropism assay with a geno2pheno FPR cut-off set at 10%, did not increase the risk of failure in this study. This finding, along with those from the MARCH study [18], supports the use of a genotypic tropism assay on DNA for selecting virologically suppressed patients to maraviroc.

Importantly, in line with the lack of drug resistance development, all patients with virological rebound rapidly regained virological suppression with alternative therapies, showing that effective therapeutic options were not lost in these cases. Of note, patients enrolled in this study were more heavily pretreated and had lower nadir CD4 counts as patients enrolled in bPI monotherapy studies, in more recent trials of maintenance therapy with bPI plus lamivudine as well as in the MARCH study [11, 18, 28].

As mentioned, study and continuation arm did not differ in the primary efficacy endpoint (proportion of patients free of treatment failure at 48 weeks on the per-protocol population). Indeed, more patients in the continuation arm interrupted their regimen because of toxicity. Most of these were renal or bone toxicities and were attributed to tenofovir disoproxil fumarate, a member of the NRTI class, which was discontinued in all cases. While the open-label study design could have facilitated treatment changes in the continuation arm, this did not happen in the study arm with maraviroc and boosted darunavir, showing a good tolerability of this regimen. In the subgroup of patients analyzed, BMD was improved in the study arm as compared to the continuation arm and, accordingly, markers of bone turnover were also improved. Altogether these findings suggest that the association of maraviroc 300 mg qd with boosted darunavir qd is well tolerated and prevents some NRTI-associated toxicity. Candidate patients should however be very carefully selected among those which guarantee the maximal adherence levels and those with prolonged viral suppression. Alternatively, it should be tested whether maintaining one of the less toxic NRTIs with this combination could be a virologically safer strategy [29].

The main limitation of this study is represented by the open label design, the heterogeneity of the regimens in the continuation arm and the inclusion of less than half patients than planned in the study. The reduced number of patients due to premature study discontinuation did not allow us to formally conclude on the noninferiority of our experimental regimen on the primary study outcome, although the lower 95% confidence interval bound of the difference between experimental and control arm was within the pre-specified lower margin for defining noninferior efficacy. The heterogeneity of the comparator regimens allowed to compare the efficacy of a drug reduction strategy against a number of different standard 3-drug regimens which more closely reflect the reality of currently treated and virologically controlled patients in clinical practice.

The inferior virologic efficacy of the dual regimen tested here contrasts with results obtained with other dual regimens showing non-inferior efficacy to 3-drug therapy, in particular ritonavir-boosted PI regimens combined with lamivudine and dolutegravir with rilpivirine [30–35]. Therefore, specific dual therapy approaches may be effective but MVC-based dual therapies seem virologically inferior.

In conclusion, switching a standard 3-drug therapy to maraviroc 300 mg qd plus boosted darunavir qd in virologically suppressed patients was associated with an improved tolerability but was virologically inferior as compared to continuing the previous therapy. Virological failures were associated with lower adherence, which caused suboptimal drug exposure. Genotypic tropism testing on viral DNA can be employed to select virologically suppressed patients to maraviroc but candidates to the dual therapy regimen tested here need to be carefully selected and instructed.

## Supporting information

S1 Table Baseline patients characteristics (intent-to-treat population). (DOCX)

S2 Table Baseline patients characteristics of virological failures vs non-failures in the MVC+DRV/r arm. (DOCX)

S3 Table Laboratory grade 3/4 toxicities in the ITT population. (DOCX)

S1 Text Protocollo di studio GUSTA versione 1.1 (Original Italian version). (DOC)

S2 Text GUSTA protocol versione 1.1 (English translation). (DOCX)

S3 Text CONSORT checklist. (DOC)
