# Supplementary material for: Switch to maraviroc with darunavir/r, both QD, in patients with suppressed HIV-1 was well tolerated but virologically inferior to standard antiretroviral therapy: 48-week results of a randomized trial
Source: PLoS One. 2017 Nov 21;12(11):e0187393. doi: 10.1371/journal.pone.0187393 (PMC5697828; doi:10.1371/journal.pone.0187393)
Supplement: S1 Table — (DOCX) [file pone.0187393.s001.docx]

|  | **Overall**  **n=123** | **DRV/r + MVC**  **(Arm S)**  **n=62** | **3-drug ART**  **(Arm C)**  **n=61** | **P**  **(between arms)** |
| --- | --- | --- | --- | --- |
| Age, years* | 49 (42-57) | 51 (45-59) | 48 (41-55) | 0.097 |
| Male gender | 92 (75) | 48 (77) | 44 (72) | 0.640 |
| Caucasian ethnicity | 111 (90) | 56 (90) | 55 (90) | 0.325 |
| Risk factor: |  |  |  | 0.292 |
| Heterosexual | 50 (41) | 27 (43) | 23 (38) |  |
| Homo/bisexual | 43 (35) | 22 (35) | 21 (34) |  |
| Past injecting drug user | 11 (9) | 7 (11) | 4 (7) |  |
| Other/unknown | 20 (16) | 6 (10) | 13 (21) |  |
| HCV co-infection | 16 (13) | 9 (14) | 7 (11) | 0.324 |
| Years from HIV diagnosis* | 12 (7-18) | 14 (7-19) | 11 (7-16) | 0.203 |
| Years from first ART initiation* | 10 (6-16) | 10 (6-16) | 10 (6-15) | 0.423 |
| Months from last regimen initiation* | 52 (34-69) | 55 (37-74) | 51 (30-66) | 0.198 |
| Months from last HIVRNA>50 cp/mL** | 58 (52-64) | 58 (50-67) | 58 (49-67) | 0.985 |
| CD4 nadir, cells/µL* | 209 (106-299) | 201 (87-303) | 222 (136-297) | 0.468 |
| CD4, cells/µL* | 660 (488-916) | 659 (503-1005) | 686 (482-902) | 0.754 |
| Treatment at screening: |  |  |  |  |
| NRTI | 116 (94) | 56 (90.3) | 60 (98.4) | 0.114 |
| TDF | 78 (63) | 37 (60) | 41 (67) | 0.455 |
| NNRTI | 23 (19) | 10 (16.1) | 13 (21.3) | 0.613 |
| InSTI | 22 (18) | 11 (17.7) | 11 (18) | 1.000 |
| PI | 73 (63) | 40 (64.5) | 33 (54.1) | 0.321 |
| Boosted PI | 51 (41) | 33 (53.2) | 28 (45.9) | 0.417 |
| DRV/r | 32 (26) | 15 (24.2) | 17 (27.9) | 0.642 |
| QD regimen at screening | 79 (64) | 39 (63) | 40 (37) | 0.851 |

**S 1 Table. Baseline patients characteristics (intent-to-treat population)**

Results are expressed as n (%), *median (IQR) or ** mean (95% CI)

Abbreviations legend: DRV/r, darunavir/ritonavir; MVC, maraviroc; ART, antiretroviral therapy; TDF/FTC, tenofovir/emtricitabine; HCV, hepatitis C virus; PI, protease inhibitor; NNRTI, non-nucleoside reverse transcriptase inhibitor; NRTI, nucleoside reverse transcriptase inhibitor; InSTI, Integrase strand transfer inhibitors; QD once daily.
