# Supplementary material for: Switch to maraviroc with darunavir/r, both QD, in patients with suppressed HIV-1 was well tolerated but virologically inferior to standard antiretroviral therapy: 48-week results of a randomized trial
Source: PLoS One. 2017 Nov 21;12(11):e0187393. doi: 10.1371/journal.pone.0187393 (PMC5697828; doi:10.1371/journal.pone.0187393)
Supplement: S2 Table — (DOCX) [file pone.0187393.s002.docx]

**S 2 Table. Baseline patients characteristics of virological failures vs non-failures in the MVC+DRV/r arm**

|  | **Patients with virological failure**  **(n=8)** | **Patients without virological failure**  **(n=54)** | **P-value**  **(between groups)** |
| --- | --- | --- | --- |
| FPR* | 66 (45-95) | 38 (20-60) | 0.013 |
| CD4 nadir, cells/µL* | 281 (59-592) | 200 (92-285) | 0.515 |
| CD4, cells/µL* | 640 (546 -1008) | 664 (490-999) | 0.665 |
| Years from HIV diagnosis* | 9 (7-13) | 15 (7-20) | 0.257 |
| Years from first ART initiation* | 9 (6 -12) | 10 (6-16) | 0.276 |
| Months from last HIV-RNA >50 cp/mL** | 37 (16-58) | 61 (52-71) | 0.057 |
| Months from last regimen initiation* | 46 (33-72) | 55 (37-74) | 0.738 |
| Treatment at screening: |  |  |  |
| NRTI | 8 (100) | 48 (89) | 1.000 |
| NNRTI | 0 | 10 (18) | 0.333 |
| TDF | 6 (75) | 30 (55) | 0.450 |
| InSTI | 1 (12) | 10 (18) | 1.000 |
| PI | 7 (87) | 33 (61) | 0.240 |
| bPI | 6 (75) | 27 (50) | 0.174 |
| DRV/r | 4 (50) | 11 (20) | 0.088 |
| HCV co-infection | 2 (25) | 7 (13) | 0.328 |
| Baseline HIV-1 RNA undetectable | 4 (50) | 38 (74) | 0.418 |

Results are expressed as n (%), * median (IQR) or **mean (95% CI)

Abbreviations legend: DRV/r, darunavir/ritonavir; MVC, maraviroc; FPR, false positive rate; ART, antiretroviral therapy; TDF/FTC, tenofovir/emtricitabine; HCV, hepatitis C virus; PI, protease inhibitor; NNRTI, non-nucleoside reverse transcriptase inhibitor; NRTI, nucleoside reverse transcriptase inhibitor; InSTI, Integrase strand transfer inhibitors.
