# Supplementary material for: Switch to maraviroc with darunavir/r, both QD, in patients with suppressed HIV-1 was well tolerated but virologically inferior to standard antiretroviral therapy: 48-week results of a randomized trial
Source: PLoS One. 2017 Nov 21;12(11):e0187393. doi: 10.1371/journal.pone.0187393 (PMC5697828; doi:10.1371/journal.pone.0187393)
Supplement: S3 Table — (DOCX) [file pone.0187393.s003.docx]

**S 3 Table. Laboratory grade 3/4 toxicities in the ITT population**

| **Definition grade 3/4 toxicity** | | **DRV/r + MVC**  **(Arm S)**  **n=62** | **3-drug ART**  **(Arm C)**  **n=61** | **P** |
| --- | --- | --- | --- | --- |
| Total cholesterol | >300 mg/dL | 2 (3.2%) | 2 (3.3%) | 1.000 |
| LDL cholesterol | >190 mg/dL | 4 (6.5%) | 7 (11.5%) | 0.363 |
| Triglycerides | >750 mg/dL | 2 (3.2%) | 5 (8.2%) | 0.273 |
| Creatinine | >2,2 mg/dL | 1 (1.6%) | 1 (1.6%) | 1.000 |
| Total bilirubin | >3 mg/dL | 3 (4.8%) | 4 (6.6%) | 0.717 |
| Lipases | >190 mg/dL | 2 (3.2%) | 2 (3.3%) | 1.000 |
| Amylases | >200 UI/L | 1 (1.6%) | 0 | 1.000 |
| Creatin Kinase | >1680 UI/L | 0 | 2 (3.3%) | 0.244 |
| Glucose | >250 mg/dL | 2 (3.2%) | 0 | 0.496 |
| Uric acid | >12 mg/dL | 6 (9.7%) | 5 (8.2%) | 1.000 |
| Calcium | <7 mg/dL  >12.5 mg/dL | 0  0 | 2 (3.3%)  1 (1.6%) | 0.244  0.496 |
| Phosphatum | <2 mg/dL | 14 (22.6%) | 15 (24.6%) | 0.834 |
| Potassium | >6.5 mEq/L | 4 (6.5%) | 1 (1.6%) | 0.365 |
| Sodium | <125 mEq/L | 1 (1.6%) | 0 | 1.000 |
| Neutrophil count | <750 cell/mm3 | 0 | 1 (1.6%) | 0.496 |
| At least one laboratory AE |  | 28 (45.2%) | 24 (42.3%) | 0.638 |

Abbreviations legend: DRV/r, darunavir/ritonavir; MVC, maraviroc; ART, antiretroviral therapy; AE, adverse event.
