# Supplementary material for: Switch to maraviroc with darunavir/r, both QD, in patients with suppressed HIV-1 was well tolerated but virologically inferior to standard antiretroviral therapy: 48-week results of a randomized trial
Source: PLoS One. 2017 Nov 21;12(11):e0187393. doi: 10.1371/journal.pone.0187393 (PMC5697828; doi:10.1371/journal.pone.0187393)
Supplement: S1 Text — (DOC) [file pone.0187393.s004.doc]

**Studio multicentrico randomizzato sulla sicurezza ed efficacia dello switch terapeutico a maraviroc + darunavir/ritonavir in singola somministrazione quotidiana in pazienti che effettuano almeno tre farmaci antiretrovirali appartenenti ad almeno una delle 3 classi storiche e presentano un controllo virologico ottimale (GUided Simplification with Tropism Assay, GUSTA Study).**

**Razionale dello studio.**

La maggior parte dei pazienti in terapia antiretrovirale moderna raggiunge livelli non rilevabili di carica virale. Oltre l’85% dei pazienti negli studi di coorte presenta una carica virale (HIV RNA plasmatico) inferiore alle 50 copie/mL. Sei classi di antiretrovirali sono attualmente registrate in Italia. Tra di esse, ultima introdotta nel commercio, la classe degli antagonisti del corecettore CCR5, impiegato per l’ingresso dei virus a tropismo R5. Il maraviroc (MVC) , primo membro approvato di tale classe, si è dimostrato efficace nei pazienti in fallimento virologico [Motivate NEJM; PMID 20703158 (96-wk)]. Il sequenziamento ottimale delle classi antiretrovirali è presupposto fondamentale per un’efficacia a lungo termine del trattamento. Un sequenziamento terapeutico corretto deve tenere conto del risparmio delle classi per il loro uso futuro. Tuttavia, per la classe degli antagonisti del CCR5 si pone il problema che il loro impiego più ritardato aumenta la probabilità che sia presente o si selezioni un virus in grado di utilizzare un corecettore alternativo (CXCR4), quindi insensibile a tali farmaci. Tale classe, il cui impiego è subordinato ad una verifica del tropismo virale su virus plasmatico (che deve necessariamente essere R5-tropico), non è per il momento approvata in prima linea terapeutica in Europa ed il suo impiego nei pazienti virologicamente soppressi (HIV RNA <50 copie/mL) non è praticabile per l’impossibilità di valutare il tropismo virale in assenza di virus circolante rilevabile. Pertanto il loro impiego può essere tentato esclusivamente in pazienti in fallimento virologico, quando la probabilità che il virus abbia un tropismo esclusivamente R5 è ridotta.

Il tropismo virale può essere determinato con metodi fenotipici (che di norma impiegano virus ricombinante) o genotipici. L’unico saggio clinicamente validato in studi prospettici è il Trofile di prima generazione [cit Motivate]. Tale saggio, che consentiva il rilevamento dei ceppi X4 se costituenti almeno il 10% della popolazione virale plasmatica, è stato successivamente sostituito dal Trofile ES, più sensibile nella rilevazione dei ceppi X4 (fino a 0.3% delle quasispecie). Recentemente diversi sistemi interpretativi sono stati messi a punto allo scopo di predire il tropismo virale a partire dalla sequenza del dominio V3 della glicoproteina di superficie gp120, maggiore determinante del tropismo corecettoriale. I più impiegati, liberamente disponibili in rete, sono il geno2pheno[coreceptor] ed il PSSM. La concordanza fra il tropismo predetto su base genotipica e quello ottenuto su base fenotipica è sensibilmente migliorata con i sistemi genotipici di ultima generazione, soprattutto quando, oltre alle informazioni sulla sequenza, vengono inserite nel sistema predittivo delle variabili cliniche [Prosperi, retrovirology 2010]. Sia il Trofile ES che i sistemi basati sul genotipo, sono stati validati clinicamente in maniera retrospettiva [Merit Journal of Infectious Diseases, 2010, Harrigan PMID 20736814]. L’interpretazione genotipica è stata anche validata prospetticamente, sia pure se in studi ancora numericamente limitati [tedeschi]. Diversi studi hanno infine analizzato la correlazione tra interpretazione genotipica a partire dal DNA virale da campioni di PBMC o di sangue intero e quella a partire da RNA plasmatico, individuando una concordanza uguale o superiore all’85% [Prosperi, Retrovirology 2010; PMID 20488982]. Si ritiene pertanto che l’indagine del tropismo mediante sequenziamento del DNA virale possa essere idonea per guidare lo switch terapeutico ad antagonisti del CCR5 in pazienti con soppressione virologica. Dati preliminari da studi spontanei non controllati sembrano indicare che lo switch a maraviroc, il primo antagonista del CCR5 disponibile nel commercio, sia associato a mantenimento della soppressione virologica [Geretti AM, personal communication]. La prova definitiva circa la sicurezza ed efficacia di tale strategia potrà avvenire esclusivamente attraverso uno studio clinico randomizzato.

Maraviroc è stato approvato ad un dosaggio di 300 mg bid: esso va ridotto a 150 mg bid quando combinato con un PI potenziato nella farmacocinetica da ritonavir (PI/r); in assenza di PI/r ed in presenza di farmaci NNRTI che ne riducano l’esposizione, esso va impiegato a 600 mg bid. Il dosaggio bid è stato preferito al OAD in virtù di una minima superiorità osservata negli studi di fase III Motivate, in pazienti plurifalliti alle tre classi storiche di farmaci. Nelle analisi dei sottogruppi di pazienti, l’efficacia di maraviroc OAD è risultata molto simile a quella del bid e la raccomandazione del dosaggio BID si basa sull’osservazione della sua superiorità a 24 settimane esclusivamente nel sottogruppo di pazienti che al basale presentava bassissimi CD4 (<50) e viremia plasmatica >100,000 copie/mL o attività del background regimen pari a 0 [Gulick RM, van der Ryst E, Lampiris H, et al. Efficacy and safety of once-daily (QD) compared with twice-daily (BID) maraviroc plus optimized background therapy (OBT) in treatment-experienced patients infected with CCR5-tropic-HIV-1: 24-week combined analysis of the MOTIVATE 1 and 2 studies. Presented at the 4th IAS Conference on HIV Pathogenesis, Treatment and Prevention, Sydney, July 22–25, 2007. abstract.]. Pertanto è assai verosimile attendersi un’efficacia sovrapponibile della somministrazione OAD di MVC in un contesto di semplificazione terapeutica, a soppressione virologica già avvenuta.

Darunavir/ritonavir 800/100 mg OAD è uno dei terzi farmaci raccomandati per l’impiego in prima linea terapeutica. Uno studio clinico randomizzato di fase III ne ha dimostrato la non-inferiorità virologica nei confronti di lopinavir/ritonavir [Artemis AIDS 2009] nei pazienti naive, con minori effetti collaterali a livello gastrointestinale ed un minore impatto sui lipidi ematici. La medesima posologia di darunavir/r OAD ha dimostrato la non-inferiorità rispetto a darunavir/r BID in pazienti experienced in fallimento terapeutico, benché il basso numero di pazienti con resistenza ai PI in tale studio ne limitasse la portata [Cahn P, CROI 2010, abs 57]. L’elevata barriera genetica del darunavir è dimostrata dall’assenza di mutazioni di resistenza al fallimento in prima linea e dalla superiorità virologica rispetto a lopinavir nei pazienti PI-experienced con minore selezione di mutanti resistenti nei pazienti in fallimento virologico [Titan]. Tale elevata barriera genetica ne ha consentito l’impiego in semplificazione terapeutica con monoterapia in pazienti virologicamente soppressi senza precedenti fallimenti ai PI [Monoi, Katlama C AIDS 2010]; il dosaggio OAD in monoterapia è risultato non-inferiore a 48 settimane rispetto alla triplice terapia in pazienti senza precedenti fallimenti [Monet Arribas J AIDS 2010], ma non a 96 settimane [Monet XVIII AIDS Conference Vienna 2010], quando la non-inferiorità rispetto al braccio in triplice terapia era mantenuta solo non considerando falliti i pazienti del braccio monoterapia che riprendevano i 2 NRTI.

Lo studio della associazione darunavir/r OAD + maraviroc OAD nasce pertanto dall’esigenza di coniugare terapie NRTI-sparing a basso impatto metabolico e con minori effetti collaterali con strategie in grado di assicurare una costante efficacia virologica anche in pazienti precedentemente falliti e con possibili resistenze di classe, che possiedano una buona penetrazione nei santuari anatomici (SNC e genitale). Le semplificazioni terapeutiche in pazienti con viremia controllata, con riduzione del numero di farmaci impiegati, sono attualmente oggetto di numerosi studi che hanno l’obiettivo di risparmiare tossicità e costi. Sono in corso di studio nuove associazioni di biterapie in prima linea terapeutica (come LPV/r+ RAL, studio Progress XVIII AIDS Conference Vienna 2010, o ATV unboosted con RAL, studio SPARTAN XVIII AIDS Conference Vienna 2010) che tuttavia, a fronte di un’ottima tollerabilità e buona efficacia osservate negli studi pilota, non permettono somministrazioni QD e sono gravate da costi pesanti causati dalla necessità di aumentare alcune dosi di farmaco o dal costo di alcuni farmaci di nuova generazione. L’interazione favorevole con le bassi dosi di ritonavir impiegate nel presente schema, consentirebbe invece una somministrazione QD di una singola compressa di maraviroc da 300 mg con notevole beneficio sia in termini di praticità e convenience che in termini di impatto economico (la spesa è inferiore a quella dell’associazione fissa tenofovir/emtricitabina, uno standard impiagato in mumerose associazioni terapeutiche antiretrovirali). Recentemente sono stati presentati i risultati preliminari dello studio pilota A4001078 in pazienti naive che confrontava atazanavir/ritonavir + MVC 150 mg OAD con atazanavir/ritonavir +TDF/FTC. La posologia di MVC era dimezzata rispetto a quella consueta in virtù del doppio effetto di boosting farmacocinetico sul farmaco, esercitato sia da ritonavir che da atazanavir. Lo studio non presentava una potenza sufficiente per confronti statistici di non-inferiorità tra i due schemi, tuttavia alla settimana 24 la risposta virologica (VL<50) nel braccio ATV/r+MVC era 80%, a fronte di un 89% nel braccio ATV/r +TDF/FTC mentre l’iperbilirubinemia di grado 3/4 (indiretta) e le interruzioni per ittero erano più frequenti nel braccio con MVC (XVIII AIDS Conference Vienna 2010), che con maggiore frequenza doveva sostituire, per tale motivo, ATV/r con DRV/r. Tale effetto è probabilmente associato alla maggiore esposizione ad atazanavir nel braccio maraviroc rispetto al braccio tenofovir/emtricitabine Nel presente studio l’esposizione al MVC sarà di 300 mg OAD, tranne nei pazienti con CrCL < 80 ml/min, dove la posologia sarà ridotta a 150 mg qd, come da scheda tecnica.

**Obiettivi dello studio.**

1. Dimostrare la non-inferiorità virologica a 48 settimane della semplificazione terapeutica verso maraviroc (MVC) QD + darunavir/ritonavir (DRV/r) QD nei confronti della prosecuzione del precedente trattamento in pazienti con virus R5-tropico, che effettuano almeno 3 farmaci ARV, che presentano una tossicità di qualsiasi grado e tipo con il trattamento in atto o desiderino una semplificazione terapeutica o una interruzione proattiva di NRTI ed abbiano una viremia persistentemente soppressa nelle ultime 24 settimane.
2. Verificare e confrontare l’andamento dei parametri immunologici e di tossicità tra i due bracci di randomizzazione e l’impatto in termini farmaco economici della semplificazione proposta

**Disegno:**

Si tratta di uno studio multicentrico, prospettico, randomizzato 1:1, di confronto in aperto, di non-inferiorità. Saranno arruolati 330 pazienti consecutivi (165 per braccio). La durata prevista per lo studio comprenderà 6 mesi per l’arruolamento, 28 giorni dallo screening al basale, 96 settimane di trattamento (48 settimane per l’end-point primario) e 30 giorni di follow-up dopo la fine dello studio. In caso di fallimento virologico (definito come riscontro di 2 valori di HIV-RNA consecutivi superiori a 50 copie/mL o un singolo valore superiore a 1000 copie/mL) sarà effettuata modificazione della terapia guidata dal genotipo di resistenza ed interruzione dell’antagonista del CCR5; in caso di mancata genotipizzazione per bassi livelli di viremia (o altri motivi tecnici) il nuovo regime comprenderà almeno un PI/r associato a farmaci scelti sulla base del migliore giudizio clinico. Il farmaco sarà fornito come da routine dalle strutture di outpatients dei centri aderenti. Saranno registrati lotto e data di scadenza di ogni confezione di farmaco consegnata.

**Criteri di inclusione:**

Pazienti in terapia con almeno 3 farmaci antiretrovirali (ritonavir non considerato nel computo dei 3) immodificati da almeno 12 settimane

Di età maggiore o uguale a 18 anni

Con viremia <50 copie/mL in almeno 2 determinazioni consecutive da almeno 6 mesi (margine di tolleranza 2 settimane)

Con CD4 >200 cellule/mm3 da almeno 3 mesi ed assenza di infezioni opportunistiche maggiori attive o altre patologie AIDS-definenti per almeno un anno prima dello screening

Con tropismo virale R5 predetto sulla base dell’interpretazione della sequenza della regione V3 del DNA virale e degli altri parametri clinici secondo geno2pheno “clonal”

Che abbiano fornito il consenso informato alla partecipazione allo studio

**Criteri di esclusione:**

Gravidanza o allattamento, desiderio di gravidanza a breve termine

Presenza di patologie maggiori non AIDS-definenti che, a giudizio dell’investigatore possano compromettere la permanenza del paziente nello studio per il follow-up necessario

Presenza di almeno una mutazione di resistenza maggiore o di almeno due mutazioni di resistenza minori che possano ridurre la suscettibilità a darunavir secondo l’ultima lista aggiornata di International AIDS Society - USA, documentata nell’ultimo o in precedenti test di resistenza

Pregressa esposizione a farmaci antagonisti di CCR5.

Pregressa determinazione del tropismo su plasma indicante ceppi D/M o X4

Precedenti tossicità cliniche maggiori (grado >=3) ai farmaci oggetto dello studio (darunavir; ritonavir ad una posologia <300 mg/die) o quelli in atto

Storia di allergia ai sulfamidici

Positività di HBsAg

Cirrosi epatica con classe di Child-Pugh C

Tasso di filtrazione glomerulare stimato < 30 ml/min (Cockroft-Gaut; MDRD se razza nera africana o afro-americana) alla visita di screening

Ipertransaminasemia di grado IV (oltre 10 volte il valore superiore della norma) alla visita di screening

**Endpoint primario:**

percentuale di pazienti con fallimento virologico (definito come riscontro di 2 valori di HIV-RNA consecutivi superiori a 50 copie/mL o un singolo valore superiore a 1000 copie/mL) (TLOVR) a 48 settimane secondo l’analisi per protocol con switch=fallimento. Switch=sospensione o aggiunta di qualsiasi farmaco nel braccio MVC+DRV/r; nel braccio di controllo idem

**Endpoints secondari:**

- percentuale di pazienti con fallimento virologico (definito come riscontro di 2 valori di HIV-RNA consecutivi superiori a 50 copie/mL o un singolo valore superiore a 1000 copie/mL) a 96 settimane secondo l’analisi intention to treat con missing value=failure.
- tempo intercorso tra il baseline ed il fallimento virologico (definito come riscontro di 2 valori di HIV-RNA consecutivi superiori a 50 copie/mL o un singolo valore superiore a 1000 copie/mL) secondo analisi di sopravvivenza
- percentuale dei pazienti che presentano al fallimento virologico un RNA virale plasmatico ed un DNA provirale con tropismo predetto di tipo X4.
- evoluzione della conta dei CD4 nelle 96 settimane di studio
- evoluzione dell’aderenza auto-riportata alla terapia, della qualità della vita e dei sintomi auto-riportati dopo 24, 48 e 96 settimane di studio
- evoluzione delle concentrazioni plasmatiche di maraviroc nelle 96 settimane di studio
- evoluzione dei parametri metabolici nelle 96 settimane di studio
- impatto farmacoeconomico della associazione DRV/r+MVC in rapporto al braccio di controllo
- modifica dei risultati dei test neurocognitivi a 48 e 96 settimane (sottogruppo)
- modifica della densitometria ossea e del tessuto adiposo sottocutaneo misurati con DEXA a 48 e 96 settimane (sottogruppo)
- modifica di IMT e FMD a 48 e 96 settimane (sottogruppo)

**Calcolo del campione e metodologia statistica:**

Assumendo che proseguendo la terapia in atto il 90% rimane soppresso a 48 settimane per dimostrare la non-inferiorità di MVC+DRV/r versus la prosecuzione della HAART in corso (delta = -10%, con un intervallo di confidenza del 95% ed una potenza dell’80%), assumendo una perdita del 5% dei pazienti dallo per rispettare il criterio di analisi “per protocol”, sono richiesti 165 pazienti per braccio.

- Analisi dell’end-point primario:
- Analisi principale: Per protocol (PP): escludendo pazienti con violazioni maggiori del protocollo o randomizzati non correttamente. Time to loss of virolgical response (TLOVR)
- Analisi secondarie:
  - Observed: solo pazienti con dato virologico disponibile.
  - Intent To Treat (ITT) – tutti I pazienti randomizzati
    - Switch = Fallimento (S = F)
    - Switch ma con soppressione virologica = non fallimento (S ¹ F)

Il tempo intercorso dall’inizio del trattamento al raggiungimento dell’endpoint verrà valutato tramite analisi di sopravvivenza, le differenze nei parametri laboratoristici verrà analizzata tramite T-test per campioni indipendenti o test esatto di Fisher al baseline, test per campioni appaiati (per confrontare modificazioni dei parametri rispetto al baseline).

**Procedure dello studio:**

**Valutazione preliminare (visita di screening):**

Durante la visita di screening sarà ottenuto il consenso informato scritto del paziente, raccolta la storia clinica e terapeutica (antiretrovirale e non) completa con registrazione di tutte le terapie concomitanti, ricerca dei marcatori di infezione da HBV e da HCV, test di gravidanza per le donne in età fertile o dosaggio dell’FSH sierico per le donne in menopausa da meno di 2 anni, esame obiettivo completo di parametri vitali, peso ed altezza, esami ematochimici (funzionalità epatica e renale, glicemia a digiuno, profilo lipidico completo, amilasi, Na, K, P, Ca, Cl, CK, acido urico), esame emocromocitometrico completo, esame urine completo, tipizzazione linfocitaria, HIV-RNA. Verrà inoltre determinato il tropismo genotipico mediante sequenziamento di HIV-V3 dal DNA virale estratto da sangue intero.

**Visita basale:**

Sarà definito come basale (giorno 0) il giorno in cui verrà somministrata per la prima volta la terapia semplificata con maraviroc e darunavir/ritonavir. Saranno ripetuti l’esame obiettivo completo dei parametri vitali, peso ed altezza, esami ematochimici (funzionalità epatica, comprese ALT, AST, bil tot/con fosf alcalina e gammagt, e renale, glicemia a digiuno, profilo lipidico completo, amilasi, lipasi, Na, K, P, Ca, CK), l’esame emocromocitometrico completo, l’esame urine completo, la tipizzazione linfocitaria, l’HIV-RNA, e messo da parte un campione di plasma e un campione di sangue intero per l’analisi del genotipo di V3 su RNA e su DNA, rispettivamente. Saranno effettuate inoltre analisi aggiuntive relative al metabolismo osseo (fosfatasi alcalina ossea, calcitonina, paratormone, osteocalcina, dosaggio della vitamina d3, idrossiprolina urinaria). La funzionalità renale verrà monitorata tramite calcolo della CG o MDRD e la creatinina clearance, la proteinuria delle 24 ore, natriuria, kaliuria, calciuria, fosfaturia, cloruria ed uricuria delle 24 ore. Sarà inoltre valutata l’insulinemia a digiuno. In specifici sottogruppi verrà misurato tramite ecografia (Liposound) il grasso sottocutaneo a livello di volto, braccia e coscia, lo spessore intimale medio (IMT) mediante ecografia-doppler carotidea, la funzione endoteliale mediante flow-mediated dilation (FMD) dell’arteria brachiale e la densitometria ossea e la distribuzione del grasso sottocutaneo tramite DEXA. Infine, al paziente sarà richiesto di compilare un questionario sull’aderenza autoriportata, un questionario sui sintomi autoriportati e sulla qualità della vita e sarà effettuata una valutazione di base con test neurocognitivi di screening, con eventuale somministrazione di test di approfondimento in caso di esito patologico dei test preliminari.

Nella giornata i pazienti candidati saranno sottoposti a randomizzazione 1:1 nei seguenti bracci:

1. Maraviroc 300* mg (1 cp) ogni 24 h + darunavir 800 mg (2cp) ogni 24 h + ritonavir 100 mg (1 cp) ogni 24 h AL MATTINO (allo scopo di rendere possibile il TDM)
2. Prosecuzione della precedente terapia in atto

***150 mg (1 cp) ogni 24 h in caso di clearance della creatinina stimata <80 ml/min**

**Monitoraggio:**

Le visite previste dopo il basale saranno a 4, 12, 24, 36, 48, 60, 72, 84 e 96 settimane.

Ad ogni visita saranno monitorati gli esami ematochimici (tutti i lipidi, transaminasi, bilirubina tot/con, amilasi creatinina, na, k, P, CK, glicemia a digiuno, insulinemia al basale e a 48 settimane sempre a digiuno) ed emocromocitometrico, l’analisi delle urine, la tipizzazione linfocitaria e la viremia di HIV, il monitoraggio della concentrazione plasmatica di maraviroc, darunavir e ritonavir a 24+/- 2h (NB i pazienti del braccio A dovranno essere istruiti di non assumere i farmaci in studio prima del prelievo).

Il monitoraggio della funzionalità renale con CG o MDRD sarà ripetuto a tutte le visite mentre la creatinina clearance, la proteinuria delle 24 ore, natriuria, kaliuria, calciuria, fosfaturia, cloruria ed uricuria delle 24 ore saranno misurate alla settimana 24, 48 72 e 96.

La valutazione dell’insulinemia a digiuno e le analisi relative al metabolismo osseo (fosfatasi alcalina ossea, calcitonina, paratormone, osteocalcina, vitamina d, calciuria e fosfaturia delle 24 ore, idrossiprolina urinaria) saranno ripetute a 24, 48 e 96 settimane, la misurazione del grasso sottocutaneo a livello di volto, braccia, coscia e lo spessore intimale medio (IMT) e la funzione endoteliale mediante dilatazione flusso-mediata dell’arteria brachiale tramite ecografia e la DEXA per la densitometria ossea e per la valutazione del grasso sottocutaneo saranno invece ripetute a 48 e 96 settimane.

Il questionario sull’aderenza autoriportata verrà ripetuto a ciascuna visita, quelli sui sintomi autoriportati e sulla qualità della vita saranno somministrati a 4, 24, 48 e 96 settimane, mentre i test di valutazione neurocognitiva (batteria di screening ed eventuale approfondimento per test patologici) saranno ripetuti alla settimana 48 e 96.

In caso di fallimento virologico (riscontro di 2 valori di HIV-RNA consecutivi superiori a 50 copie/mL o un singolo valore superiore a 1000 copie/mL) sarà effettuato il test genotipico di resistenza su plasma entro un mese dall’osservazione del fenomeno. Sarà inoltre messo da parte un campione di plasma per il genotipo di resistenze e V3 di HIV e un campione di sangue intero per l’analisi su DNA virale ad ogni visita per eventuali valutazioni successive secondo il giudizio del medico responsabile dello studio.

Il diagramma riassuntivo delle visite è fornito in allegato al documento (allegato 1).

Gli esami ematochimici, ematologici e microbiologici previsti dallo studio saranno eseguiti tramite routine clinica e non comporteranno costi aggiuntivi per la struttura.

Il monitoraggio del dosaggio plasmatico del farmaco sarà eseguito in collaborazione con l’Istituto di Farmacologia (servizio di Farmacologia clinica) del Policlinico Gemelli.

La DEXA per la densitometria ossea e per la distribuzione del grasso corporeo la misurazione di IMT e di FMD saranno eseguite presso alcuni centri selezionati tra quelli partecipanti allo studio. Tali procedure non comporteranno costi aggiuntivi per la struttura.

Le terapie concomitanti saranno verificate dal medico responsabile dello studio ad ogni visita; qualsiasi modifica al trattamento in corso alla visita di screening rilevata nelle visite di monitoraggio sarà registrata.

**Visite non programmate:**

Il medico potrà richiamare il paziente per una visita non programmata ogni volta che ciò sia ritenuto necessario per la sicurezza del paziente, in particolare in relazione ad eventuali anomalie di laboratorio o eventi avversi.

Nel caso in cui in una qualsiasi delle visite la viremia risulti superiore a 50 copie/mL il paziente dovrà essere richiamato non appena il medico verrà a conoscenza del risultato e comunque ad una distanza non superiore ad 1 mese dal precedente prelievo per ripetere la viremia di HIV ed eseguire esame genotipico, dosaggio plasmatico del farmaco e valutazione approfondita dell’aderenza.

**Criteri per l’uscita dallo studio:**

Fallimento virologico, definito come riscontro di 2 valori di HIV-RNA consecutivi superiori a 50 copie/mL o un singolo valore superiore a 1000 copie/mL.

Diagnosi di infezione opportunistica o di altra patologia maggiore HIV-relata o evento avverso grave correlato con i farmaci somministrati al paziente

Anomalie di laboratorio di grado 4 agli esami di monitoraggio (ad eccezione dei lipidi)

Violazioni maggiori del protocollo

Ritiro del consenso informato

##### Uscita dallo studio

La motivazione e la data dell’uscita dallo studio dovranno essere segnalate sulla cartella clinica di ogni paziente.

I soggetti partecipanti allo studio sono liberi di recedere da tale partecipazione in ogni momento senza dovere alcuna spiegazione e senza pregiudizio alcuno nei confronti di eventuali e/o future cure a cui dovessero ricorrere nel Centro Clinico presso cui vengono seguiti.

Inoltre i soggetti possono essere fatti uscire dallo studio in qualunque momento a discrezione del medico sperimentatore, se la partecipazione allo studio rappresenta un rischio di qualsiasi natura per la salute del paziente.

**Dichiarazione di Helsinki e comitato etico**

Lo studio verrà condotto in accordo con i principi della dichiarazione di Helsinki. Il protocollo ed il foglio informativo/consenso informato per il paziente dovranno essere approvati dal Comitato Etico prima che lo studio inizi ad arruolare pazienti afferenti al Centro Clinico stesso.

Il medico sperimentatore ha la responsabilità di informare il proprio Comitato Etico di ogni Evento Avverso e di ogni emendamento al protocollo secondo quanto previsto dai decreti legislativi correnti tenendone copia nei propri archivi.

**Consenso informato dei pazienti**

Il medico sperimentatore ha la responsabilità di dare al paziente una adeguata informazione circa gli scopi, i benefici ed i possibili rischi dello studio. Il medico sperimentatore è inoltre responsabile di ottenere il consenso scritto del paziente prima di includerlo nello studio (presentato separatamente al Comitato Etico).

##### Fonti dei dati e cartelle cliniche

Lo sperimentatore ha la responsabilità di conservare i dati in originale relativi allo studio nonché la lista dei nomi e degli indirizzi dei pazienti inclusi ed il consenso informato firmato per 15 anni.

Per ogni paziente incluso dovrà essere compilata in italiano una cartella clinica la cui accuratezza ed attendibilità di compilazione saranno attestate dalla firma dello sperimentatore responsabile. Le correzioni saranno eseguite cancellando con una linea il dato sbagliato e riscrivendo il valore corretto accanto con la data e la controfirma del responsabile. Non è ammesso l'uso di correttori fluidi.

Lo sperimentatore responsabile garantirà che i partecipanti allo studio abbiano l'adeguato addestramento e che ogni informazione rilevante per la buona riuscita dello studio sia comunicata ai co-investigatori coinvolti.

**Esami virologici**

La quantificazione della carica virale plasmatica è eseguita presso i centri partecipanti mediante il sistema certificato in uso. I test genotipici per la valutazione del tropismo corecettoriale e per la resistenza ai farmaci antiretrovirali sono eseguiti centralmente presso il Dipartimento di Biologia Molecolare dell’Università di Siena su DNA ottenuto da sangue intero citratato o su RNA ottenuto da plasma. I campioni di sangue citratato possono essere conservati ed inviati a -20°C, i campioni di plasma devono essere conservati a -70°C ed inviati in ghiaccio secco. Ogni campionamento di plasma deve essere fornito in due aliquote da 1-2 ml ciascuna in provette da 1.5-2.0 ml con tappo a vite. Ogni campionamento di sangue intero deve essere fornito in singola aliquota da 1-2 ml in provetta da 1.5-2.0 ml con tappo a vite.

Le procedure di estrazione di DNA e di estrazione di RNA sono eseguite mediante cromatografia su colonna utilizzando kit commerciali (es. Qiagen). Il sequenziamento di una regione della gp120 di circa 500 basi comprendente il dominio V3 (screening su DNA, baseline su DNA ed eventualmente RNA, analisi al fallimento su RNA) è eseguito mediante procedura home made (Prosperi, Retrovirology 2010) su ABI 3130XL. Il sequenziamento delle regioni proteasi e trascrittasi inversa (analisi al fallimento su RNA) è eseguito mediante il sistema Viroseq su ABI 3130XL. Per la stima del tropismo corecettoriale si adotta il sequenziamento in triplicato seguito dall’interpretazione con geno2pheno[coreceptor] utilizzando un False Positive Rate del 10% (European guidelines for tropism testing, Lancet Infectious Diseases, in press).

##### Centri partecipanti (adesioni ottenute al 8/11/2010)

Clinica delle Malattie Infettive, Università Cattolica del Sacro Cuore, Roma

Malattie Infettive 2, Azienda Ospedaliera Universitaria Senese

Malattie Infettive 1, Azienda Ospedaliera Universitaria Senese

Clinica delle Malattie Infettive, Università di Brescia

Clinica delle Malattie Infettive, Università di Perugia

Clinica delle Malattie Infettive, Università di Firenze

Clinica delle Malattie Infettive Universitarie, Università di Milano, ospedale L Sacco

Clinica delle Malattie Infettive Ospedaliere, Ospedale L Sacco, Milano

Clinica delle Malattie Infettive, Policlinico “S Gerardo”, Monza

Clinica delle Malattie Infettive, Università di Modena

Clinica delle Malattie Infettive, Ospedale Careggi, Firenze

Clinica delle Malattie Infettive, Ospedale S. Maria Annunziata, Firenze

Clinica delle Malattie Infettive, Università di Chieti

Clinica delle Malattie Infettive, Policlinico di Rovigo

Clinica delle Malattie Infettive, Azienda Ospedaliera di Grosseto

Clinica delle Malattie Infettive, Università di Ancona

Clinica delle Malattie Infettive, Ospedale San Martino, Genova

##### Riferimenti bibliografici.

1. Palella FJ, Delaney KM, Moorman AC, et al. *Declining morbidity and mortality among patients with advanced human immunodeficiency virus infection. HIV Outpatient Study Investigators.* New England Journal of Medicine 1998; 338: 853-860
2. *Guidelines for the Use of Antiretroviral Agents in HIV-1-Infected Adults and Adolescents* Developed by the DHHS Panel on Antiretroviral Guidelines for Adults and Adolescents – A Working Group of the Office of AIDS Research Advisory Council (OARAC), December 1st, 2009, http://aidsinfo.nih.gov
3. Clumeck N, Pozniak A, Raffi F, and the European Aids Clinical Society (EACS) Executive Committee, *Guidelines for the Clinical Management and Treatment of HIV Infected Adults in Europe 2009*, version 5.2 http://www.europeanaidsclinicalsociety.org/guidelinespdf/1_Treatment_of_HIV_Infected_Adults.pdf
4. BG Gazzard on behalf of the BHIVA Treatment Guidelines Writing Group*, British HIV Association guidelines for the treatment of HIV-1-infected adults with antiretroviral therapy 2008, HIV Medicine (2008), 9, 563–608
5. Linee Guida italiane sull'utilizzo degli antiretrovirali nelle persone con infezione HIV. http://www.salute.gov.it/hiv/newsHiv.jsp?id=1114&menu=inevidenza&lingua=italiano

**ALLEGATO 1. Diagramma di flusso riassuntivo delle visite e procedure dello studio**

| **Procedura** | **Screening**  **(giorno -28)** | **Basale**  **(giorno 0)** | **Visite successive (sett. 4, 12, 36)** | **Settimana 24** | **Sett. 48** | **Sett. 60, 72, 84** | **Sett. 96** | **Visita di follow-up (a 30 gg)** |
| --- | --- | --- | --- | --- | --- | --- | --- | --- |
| Consenso informato | **X** |  |  |  |  |  |  |  |
| Storia clinica completa | **X** |  |  |  |  |  |  |  |
| Storia terapeutica completa | **X** |  |  |  |  |  |  |  |
| Farmaci concomitanti | **X** | **X** | **X** | **X** | **X** | **X** | **X** | **X** |
| HBsAg ed anti-HCV | **X** |  |  |  |  |  |  |  |
| Test di gravidanza o FSH sierico | **X** |  |  |  |  |  |  |  |
| Parametri vitali, peso, altezza, esame obiettivo completo | **X** | **X** | **X** | **X** | **X** | **X** | **X** | **X** |
| Esami ematochimici ed esame urine | **X** | **X** | **X** | **X** | **X** | **X** | **X** | **X** |
| Esami sulle urine delle 24 ore |  | **X** |  | **X** | **X** | **X****** | **X** |  |
| Esame emocromocitometrico | **X** | **X** | **X** | **X** | **X** | **X** | **X** | **X** |
| Tipizzazione linfocitaria ed HIV-RNA | **X** | **X** | **X** | **X** | **X** | **X** | **X** | **X** |
| TDM di darunavir, ritonavir e maraviroc (braccio a)*** |  |  | **X** | **X** | **X** | **X** | **X** | **X** |
| Campione di sangue intero per genotipo DNA virale | **x** | **X** | **X** | **x** | **x** | **x** | **x** |  |
| Campione per Genotipo di HIV su plasma per resistenze e tropismo | **(*)** | **(*)** | **(*)** | **(*)** | **(*)** | **(*)** | **(*)** |  |
| Insulinemia a digiuno, analisi relative al metabolismo osseo |  | **X** |  | **X** | **X** | **X** | **X** |  |
| **Valutazione ecografica grasso sottocutaneo, IMT e FMD |  | **X** |  |  | **X** |  | **X** |  |
| **DEXA massa grassa e osso |  | **X** |  |  | **X** |  | **X** |  |
| Questionario aderenza |  | **X** | **X** | **X** | **X** | **X** | **X** |  |
| Questionario sintomi autoriportati e QoL |  | **X** | **X (solo sett 4)** | **X** | **X** | **X** | **X** |  |
| **Test neuro cognitivi |  | **X** |  |  | **X** |  | **X** |  |

(*) campione conservato e stoccato a -20°C e da analizzare in caso di fallimento virologico (riscontro di 2 valori di HIV-RNA consecutivi superiori a 50 copie/mL o un singolo valore superiore a 1000 copie/mL)

** nei centri aderenti ai sottostudi relativi.

*** in un sottogruppo di 20 pazienti curva completa di farmacocinetica a 4 settimane con 9 punti di determinazione

**** solo alla settimana 72 di questa colonna
