# Supplementary material for: Switch to maraviroc with darunavir/r, both QD, in patients with suppressed HIV-1 was well tolerated but virologically inferior to standard antiretroviral therapy: 48-week results of a randomized trial
Source: PLoS One. 2017 Nov 21;12(11):e0187393. doi: 10.1371/journal.pone.0187393 (PMC5697828; doi:10.1371/journal.pone.0187393)
Supplement: S2 Text — (DOCX) [file pone.0187393.s005.docx]

**Multicenter randomized study on safety and efficacy of therapeutic switch to maraviroc + darunavir/ritonavir QD in patients** **with "standard" combination antiretroviral therapy including 3 drugs with R5 tropism by viral DNA genotyping and suppressed viremia (GUided Simplification with Tropism Assay, GUSTA Study).**

**Background and Rationale**

Most of the patients currently receiving combination antiretroviral therapy (cART) reaches undetectable levels of HIV-RNA (below 50 copies/mL in 85% in the most recent clinical trials). Currently, 6 classes of antiretroviral drugs are approved for clinical use in Italy. Among these, a CCR5 antagonist, which inhibits the viral entry in patients with CCR5-tropic virus, was the last to be introduced. Maraviroc (MVC), the first drug in this class, was proven to be efficacious in salvage therapies of patients with treatment failure [Motivate NEJM; PMID 20703158 (96-wk)]. A correct sequencing of antiretrovirals is mandatory for a long-term efficacy and should consider the need to spare drugs and drug classes for future use. Nevertheless, delaying the use of CCR5 antagonist increases the possibility to select viral strains with alternative tropism (CXCR4), and thus insensitive to these agents. So, the use of this drugs is only recommended after a tropism test confirming that the patient harbors a replicating R5-tropic virus in plasma; it is not approved for first-line use in Europe and its use in simplification strategies in virologically suppressed patients (HIV RNA <50 copie/mL) is limited because viral tropism can’t be evaluated at undetectable plasma viral loads. So, the use of CCR5 is now limited to patients experiencing treatment failure, when the probability to harbor a R5-tropic virus is lower. Viral tropism can be determined with phenotypic or genotypic tests. The only assay prospectively validated in clinical trials is the 1st generation Trofile [Motivate, see above]. This assay, with an ability to detect X4 strains present as at least 10% of the plasma viral population, was later substituted by an enhanced sensitivity version (Trofile ES), which detects X4 strains up to 0.3% of the quasispecies present in the sample. Recently, various tools have been developed in order to predict the viral tropism based on the sequence of the V3 loop of the surface glycoprotein gp120, the major determinant of viral tropism and the most used among these are geno2pheno[coreceptor] and PSSM. The concordance of the prediction with genotypic and phenotypic tools has improved, mostly when clinical variables are added to the interpretation system of genotypic results [Prosperi, retrovirology 2010]. Both, Trofile ES and genotypic assays were retrospecively validated [Merit JID, 2010, Harrigan PMID 20736814] and genotypic assays were prospectively validated in small groups of patients by German researchers. Genotypic tests using proviral DNA from PBMC were also compared to those using plasma viral RNA and found a concordance ≥ 85% [Prosperi, Retrovirology 2010; PMID 20488982]. Thus, we believe that the genotypic determination of viral tropism based on proviral DNA may be used in order to guide treatment switch to CCR5 antagonists in patients with virological suppression. Preliminary data from spontaneous uncontrolled trials seem to confirm that the treatment switch to MVC is associated with persistent virological suppression [Geretti AM, personal communication], but the final word about the safety and efficacy of this strategy may only rely on a randomized controlled trial. Maraviroc was approved at a dose of 300 mg bid, to be reduced to 150 mg bid when given in association to a boosted PI (PI/r) or increased to 600 mg bid if coadministered with NNRTIs and without PIr. The bid dosage was chosen over OAD based on a minimal superiority in the phase III Motivate study, where patients experiencing multiple treatment failures were enrolled. In subgroup analysis, the efficacy of MVC OAD was similar to MVC bid and the superiority of bid was only observed at 24w in the subgroup of patients with low baseline CD4 (<50) and high plasma viremia (>100,000 copies/mL) or absence of activity of the background regimen [Gulick RM, van der Ryst E, Lampiris H, et al. Efficacy and safety of once-daily (QD) compared with twice-daily (BID) maraviroc plus optimized background therapy (OBT) in treatment-experienced patients infected with CCR5-tropic-HIV-1: 24-week combined analysis of the MOTIVATE 1 and 2 studies. Presented at the 4th IAS Conference on HIV Pathogenesis, Treatment and Prevention, Sydney, July 22–25, 2007. abstract.]. So it is reasonable to expect a similar efficacy of MVC OAD in a setting of treatment switch in patients with suppressed viral load. Darunavir/ritonavir 800/100 mg OAD is recommended as a “third drug” in first-line cART based on a randomized clinical trial which proved its noninferior efficacy versus lopinavir/ritonavir [Artemis AIDS 2009] in naïve patients, with lower gastrointestinal side effects and lower metabolic impact. The same dosage of darunavir/r OAD was non-inferior versus darunavir/r BID in experienced patients with treatment failure even if a small number of patients harboring PI resistance was enrolled limiting the value of these results [Cahn P, CROI 2010, abs 57]. The high genetic barrier of this drug was proven by the absence of resistance mutations at failure and virological superiority and lower resistance selection versus lopinavir/r in PI-experienced patients with virological failure [Titan]. This allowed its use as monotherpay in patients with virological suppression with no previous failures to PI [Monoi, Katlama C AIDS 2010]; the OAD dosage as monotherapy was non-inferior vs standard triple therapy in patients without previous failures at 48w [Monet Arribas J AIDS 2010] but not at 96w, when the non-inferiority vs triple therapy was only maintained when patients restarting the 2 NRTI backbone were not considered as failures [Monet XVIII AIDS Conference Vienna 2010]. The combination of darunavir/r OAD with maraviroc OAD meets the clinical need to join a NRTI-sparing regimen with low metabolic impact and lower side effects with durable efficacy even in patients with previous treatment failures and with possible drug resistance along with a good penetration in the anatomic sanctuaries (SNC and genital compartment). Many studies are currently addressing this issues aiming at reducing toxicities and costs; new dual therapies are being studied for first-line therapies (i.e. LPV/r+ RAL, Progress study, XVIII AIDS Conference Vienna 2010, or ATV unboosted + RAL, SPARTAN study XVIII AIDS Conference Vienna 2010) with partially good efficacy and tolerability but who do not allow OAD dosage and are limited by their high costs. The favorable interaction with low-dose ritonavir in the regimen we suggest would allow a QD administration of MVC 300 mg with a huge benefit in convenience and costs. Recently, the preliminary results of the pilot study A4001078 in naive patients evaluated MVC 150 mg OAD + ATVr standard dose versus TDF/FTC+ATVr; the dose of MVC was half the standard dose based on a double boosting effect from both ATV and RTV. Even if the study was not powered to test for non-inferiority, the virological response (VL<50) at 24w was 80% in the ATV/r+MVC arm vs 89% in the ATV/r +TDF/FTC arm, while grade 3/4 indirect hyperbilirubinemia and treatment interruptions for jaundice were more frequent in the MVC arm (XVIII AIDS Conference Vienna 2010), probably based on a higher exposure to ATV in the MVC arm vs TDF/FTC arm. In our study, MVC will be dosed at 300 mg OAD, except for patients with CrCL < 80 ml/min, in whom it will be reduced to 150 mg qd, according to the Summary of Product Characteristics.

**Objectives**

The specific goals of the study are

1. to verify the virological non-inferiority after 48 weeks of the treatment simplification from a "standard" combination antiretroviral therapy including 3 drugs to Maraviroc (MVC) plus Darunavir/ritonavir QD, in patients virosuppressed from 24 weeks showing any grade toxicities to ongoing treatment or willing to reduce drug or need to simplify therapy or NRTI sparing regimen
2. to collect relevant information about the safety, the immunologic and the economic impact of this strategy

**Study design**

This is a 2-arm, prospective, multicenter, open- label, 1:1 randomized controlled trial. The number of patients planned to be included is 330 (165 per arm). The expected duration of the study include 6 months for enrollment, 28 days of screening at baseline, 96 weeks of treatment (48 weeks for the primary endpoint) and 30 days of follow-up after the end of the study. In case of virological failure (defined as the occurrence of 2 consecutive HIV-RNA above 50 copies / mL or a single value greater than 1000 copies / mL) therapy will be modified guided by resistance genotype and discontinuation of the CCR5 antagonist; in case of no genotyping for low levels of viremia (or other technical reasons) the new regimen will include at least one PI / r associated with drugs selected on the basis of best clinical judgment.

Drugs will be distributed by outpatients structures and batch and expiration date of each package of medication delivered will be recorded.

**Eligible subjects**

• males and females >/= 18 years of age

• HIV infected with R5 tropism by viral DNA genotyping (geno2pheno "clonal")

• treated with the same HAART regimen including 3 drugs from at least 4 months

• with at least two viral load < 50 copies/mL in two consecutive determinations at least 6 months apart (tolerance of two weeks)

• with CD4 cell count > 200 cells/μL and without any opportunistic infection or AIDS-related disease for at least one year prior to the screening

• who gave informed consent to the participation to the study.

**Non eligible subjects**

• With at least one major or two minor mutations conferring resistance to darunavir reported in the update list of International AIDS Society - USA , in previous resistance tests. Previous determination of D/M or X4 viral tropism

• Previous major clinical toxicities (grade >=3) to the proposed drugs of the study

• Pregnancy or breast feeding, desire of pregnancy in the short term

• Past exposure to CCR5 antagonist

• HBsAg positive serostatus

• Liver cirrhosis of class C (Child-Pugh)

• Sulpha drug hypersensitivity

• The presence of major non AIDS-defining diseases that, in the opinion of the investigator, may compromise the retention of the patient in the study for the necessary follow-up period.

• Estimated glomerular filtration < 30 ml/min (Cockroft-Gaut; MDRD if black-African or african-american) at screening visit

•Hypertransaminemia of grade IV (more than 10 times the upper normal limit) at screening visit

**Primary Endpoint**

proportion of patients with virological failure (defined as two consecutive measures of HIV-RNA higher than 50 copies/mL or a single measure higher than 1000 copies/mL) within 48 weeks at per protocol analysis (TLOVR), with switch=failure. Switch=stop or drug implementation in MVC+DRV/r arm; the same in control arm

**Secondary Endpoints**

• proportion of patients with virological failure (two consecutive measures of HIV-RNA higher than 50 copies/mL or a single measure higher than 1000 copies/mL) within 96 weeks at intention-to treat analysis with missing value=failure.

• time to virological failure at survival analysis

• proportion of patients with X4 viral tropism at failure (RNA or DNA genotyping)

• proportion of patients with at failure X4viral tropism at failure (RNA or DNA genotyping)

• economic impact of DRV/r+ MVC versus current ART

• modification of IMT and FMD from baseline at 48 and 96 weeks

• modification of bone density from baseline at 48 and 96 weeks

• evolution of CD4 cell count during the 96 study weeks

• modification of metabolic parameters during 96 weeks

• modification of the results of neurocognitive tests from baseline at 48 and 96 weeks

• evolution of maraviroc plasma concentrations during the 96 weeks

• evolution of adherence and quality of life after 24, 48 and 96 weeks

**Statistical analysis and sample size calculation**

Based on the assumption that by continuing the current cART regimen 90% of patients on viralogical suppression will still has an undetectable viral load at 48 weeks, in order to test for non-inferiority of MVC+DRV/r versus maintaining the current cART regimen with the “per protocol” method (delta = -10%, 95% confidence interval, 80% power), assuming a 5% of patients lost to follow-up we will need 165 patients per arm.

- Primary end-point analysis:
- Primary analysis: Time to loss of virolgical response (TLOVR), Per protocol (PP) method: incorrectly randomized patients and patients with major protocol violations will be excluded from the analysis.
- Secondary analysis:
  - Observed: only patients with an available viral load measure will be included.
  - Intent To Treat (ITT) – all randomized patients will be included
    - Switch = Failure (S = F)
    - Switch in patients with undetectable viral load = no failure (S ≠ F)

The time to each endpoint will be evaluated by survival analysis, the differences in laboratory tests will be analyzed by T-test for independent samples or Fisher’s exact test at baseline, tests for paired samples in order to evaluate the changes in specific variables from baseline.

**Study assessment and procedures**

**Screening visit**

During screening visit will be obtained written informed consent, patients medical history collection and treatment (antiretroviral or not) with complete registration of all concomitant medications, the search for markers of HBV and HCV infection, pregnancy test for women of childbearing age or serum FSH dose for women in menopause for less than 2 years, complete physical examination of vital signs, weight and height, blood tests (liver and kidney function, fasting glucose, complete lipid profile, amylase, Na, K, P , Ca, Cl, CK, uric acid), complete blood count, complete urinalysis, lymphocyte immunophenotyping, HIV-RNA. Will also be determined the genotypic tropism of HIV-V3 from viral DNA extracted from whole blood by sequencing.

**Baseline**

The day the simplified therapy with maraviroc, darunavir / ritonavir will be administered for the first time will be defined as baseline (day 0); in the control group the baseline will be represented by the first visit after the randomization . The physical examination should be repeated along with the determination of vital signs, weight and height, blood tests (liver function, including ALT, AST, tot bil / alkaline phosphate and gammagt, and kidney, fasting glucose, complete lipid profile, amylase, lipase, Na, K, P, Ca, CK), the complete blood count, urine examination complete, the lymphocyte immunophenotyping, HIV-RNA, and set aside a plasma sample and a sample of whole blood for 'genotype analysis of V3 on RNA and DNA, respectively. Furthermore, additional analysis will be performed on the bone metabolism (alkaline phosphatase, calcitonin, parathyroid hormone, osteocalcin, dose of vitamin D3, urinary hydroxyproline, urinary hydrossiploline).

Renal function is monitored by calculation of the CG or MDRD and the measured creatinine clearance, 24 hours proteinuria, natriuria, Kaliuria, calciuria, phosphaturia, chlorides and uricuria 24 hours.
Fasting insulin levels will also be assessed at baseline. In specific subgroups subcutaneous fat on the face, arm and thigh will be measured by ultrasound (Liposound); the average intima media thickness (IMT), carotid Doppler ultrasound, endothelial function by flow-mediated dilation (FMD) of brachial artery and bone mineral density and distribution of subcutaneous fat by DEXA will also be measured.

Finally, patients will be asked to complete a self-reported adherence questionnaire, a self-reported questionnaire on symptoms and quality of life and will undergo an evaluation of basic neurocognitive screening tests, and if necessary based on the screening tests, a more in depth evaluation using extensive neurocognitive assessment batteries. On the same day candidates will be subjected to the patients randomized 1:1 in the following arms:

A) * Maraviroc 300 mg (1 tablet) every 24 h + 800 mg darunavir (2 400 mg tabs) every 24 h + ritonavir 100 mg (1 heat stable tablet) every 24 h in the morning (in order to make TDM)
B) Continuation of previously ongoing treatment

* 150 mg (1 tablet) every 24 h if estimated creatinine clearance <80 ml ​​/ min

**Monitoring**

The monitoring visits will be organized at 4, 12, 24, 36, 48, 60, 72, 84 and 96 weeks.
At each visit monitoring will include blood chemistry tests (all lipids, transaminases, tot/conjugated bilirubin, amylase creatinine, Na, K, P, CK, fasting glucose, plasma) and blood cell counts, urinalysis, lymphocyte subsets and viral load of HIV, monitoring of plasma concentrations of maraviroc, darunavir and ritonavir at 24 + / - 2h (NB patients in arm A should be instructed not to take the study medication prior to sampling).
Monitoring of renal function by MDRD or CG will be repeated at all visits, while the creatinine clearance, proteinuria of 24 hours, natriuria, Kaliur, calciuria, phosphaturia, chlorides and uricuria of 24 hours will be measured at week 24, 48 72 and 96.
The assessment of fasting insulin and markers of bone metabolism (alkaline phosphatase, calcitonin, parathyroid hormone, osteocalcin, vitamin D, calciuria and phosphaturia urinary hydroxyproline) will be repeated at 24, 48 and 96 weeks, measuring subcutaneous fat on the face, arms, thighs and the average intimal thickness (IMT) and DEXA for bone densitometry will instead be repeated at 48 and 96 weeks. The questionnaire on self-reported adherence will be repeated at each visit; the questionnaire on self-reported symptoms and quality of life will be administered to 4, 24, 48 and 96 weeks, while the assessment of neurocognitive tests (battery of screening tests for investigation and possible pathological) will be repeated at week 48 and 96.
In case of virological failure (occurrence of 2 consecutive HIV-RNA above 50 copies / mL or a single value greater than 1000 copies / mL) a genotypic resistance test on plasma viral RNA will be performed within one month from the observation. At each visit, a plasma sample for the V3 genotype and resistance to HIV and a sample of whole blood for analysis of viral DNA will be stored for possible future evaluation as judged by the principal investogator of the study. The flow chart of the visits is attached to the document (Annex 1). Blood chemistry, hematology and microbiological studies will be performed under the clinical routine and without additional costs for the structure.
The therapeutic plasmatic drug monitoring will be carried out in collaboration with the Institute of Pharmacology (Clinical Pharmacology Service) of the Gemelli Hospital.
DEXA for bone densitometry, body fat evaluation and IMT will be performed at selected centers among the sites participating. These procedures do not lead to additional costs for the structure.
The concomitant therapy will be verified by the study doctor at each visit, any changes to existing treatment at the screening visit recorded in the monitoring visits will be recorded.

**Unscheduled visits**

The investigator may call the patient for an unscheduled visit whenever it is deemed necessary for patient safety, particularly in relation to possible adverse events or laboratory abnormalities. Should at any of the visits viremia be higher than 50 copies / mL, the patient should be called as soon as the doctor will know this outcome and in any case no longer than one month after the viral load testing in order to repeat the viral load testing and run HIV resistance genotyping and plasma drug levels and perform a thorough investigation of medication adherence.

**Criteria for exit from the study**

Virological failure, defined as the occurrence of 2 consecutive HIV-RNA above 50 copies / mL or a single value greater than 1000 copies / mL.

Diagnosis of opportunistic infection or other HIV-related disease or serious adverse events associated with drugs administered to the patient

Grade 4 laboratory abnormalities at monitoring exams (except for lipids)

Major protocol violations

Withdrawal of informed consent

**Exit from the study**

Motivation and exit date from the study will be reported on the medical records of each patient.
The study participants are free to withdraw from the participation at any time without giving any explanation and without any prejudice against any and / or future treatments for which used in the Clinical Center at which they are followed.
Also, people can be excluded from the study at any time at the discretion of the investigator, if participation in the study poses any kind of risk to the health of the patient.

**Helsinki declaration and Ethic Committee**

This study will be conducted in accordance with Good Clinical practice (GCP), as defined by the International Conference on Harmonisation (ICH) and in accordance with the ethical principles underlying European Union directive 2001/20/EC. The study will be conducted in compliance with the protocol. The protocol and any amendments and the subject informed consent will receive Independent Ethics Committee (IEC) approval prior to initiation of the study. All potential serious breaches must be reported to the study coordinator immediately.

**Written informed consent**

##### The physician investigator is responsible for giving to the patient adequate information about the aims, benefits and possible risks of the study. The physician investigator is also responsible for obtaining the written consent of the patient before enrollment in the study (presented separately to the Ethics Committee).

**Data sources and medical records**

The investigator has the responsibility to preserve the original data from the study and the list of names and addresses of patients included and signed informed consents for 15 years.
For each patient included an Italian medical records will be completed, whose accuracy and reliability of the compilation will be attested by the signature of the investigator. Corrections will be made ​​by deleting a line with the erroneous data and rewrite the correct value with the next date and the countersignature of the controller. It is not allowed the use of correction fluid. The investigator will ensure that study participants have the proper training and that any information relevant to the success of the study is forwarded to the co-investigators involved.

**Virological exams**

The quantification of plasma viral load will be performed at the participating centers using the certificate system in use. The genotypic test for the assessment of viral tropism and resistance to antiretroviral drugs are performed at the Department of Molecular Biology, University of Siena or in local laboratories that use certificate system on quality test, on DNA from citrated whole blood or from plasma RNA. The citrated blood samples may be stored and sent at -20 ° C, plasma samples should be stored at -70 ° C and sent on dry ice. Each plasma sample must be supplied in two aliquots of 1.2 ml each in 1.5-2.0 ml tubes with screw cap. Each whole blood sample must be provided as a single sample of 1-2 ml in a 1.5-2.0 ml tube with screw cap.

The procedures for extraction of DNA and RNA extraction will be performed by column chromatography using commercial kits (eg Qiagen). The sequencing of a region of gp120 of about 500 base pairs comprising the V3 domain (screening samples on DNA, baseline samples on DNA and, if viremic, on RNA, the failure samples on RNA) is performed by applying a home-made technique ​​(Prosperi, Retrovirology 2010) on ABI 3130XL. The sequencing of the regions of protease and reverse transcriptase (RNA analysis at failure) is executed using the Viroseq assay on ABI 3130XL.

To estimate the viral tropism we adopt the following interpretation of sequencing in triplicate with geno2pheno [coreceptor] using a 10% False Positive Rate (European guidelines for tropism testing, Lancet Infectious Diseases).

##### Clinical centers (08/11/2010)

Clinica delle Malattie Infettive, Università Cattolica del Sacro Cuore, Roma

Malattie Infettive 2, Azienda Ospedaliera Universitaria Senese

Malattie Infettive 1, Azienda Ospedaliera Universitaria Senese

Clinica delle Malattie Infettive, Università di Brescia

Clinica delle Malattie Infettive, Università di Perugia

Clinica delle Malattie Infettive, Università di Firenze

Clinica delle Malattie Infettive Universitarie, Università di Milano, ospedale L Sacco

Clinica delle Malattie Infettive Ospedaliere, Ospedale L Sacco, Milano

Clinica delle Malattie Infettive, Policlinico “S Gerardo”, Monza

Clinica delle Malattie Infettive, Università di Modena

Clinica delle Malattie Infettive, Ospedale Careggi, Firenze

Clinica delle Malattie Infettive, Ospedale S. Maria Annunziata, Firenze

Clinica delle Malattie Infettive, Università di Chieti

Clinica delle Malattie Infettive, Policlinico di Rovigo

Clinica delle Malattie Infettive, Azienda Ospedaliera di Grosseto

Clinica delle Malattie Infettive, Università di Ancona

Clinica delle Malattie Infettive, Ospedale San Martino, Genova

##### References

1. Palella FJ, Delaney KM, Moorman AC, et al. *Declining morbidity and mortality among patients with advanced human immunodeficiency virus infection. HIV Outpatient Study Investigators.* New England Journal of Medicine 1998; 338: 853-860
2. *Guidelines for the Use of Antiretroviral Agents in HIV-1-Infected Adults and Adolescents* Developed by the DHHS Panel on Antiretroviral Guidelines for Adults and Adolescents – A Working Group of the Office of AIDS Research Advisory Council (OARAC), December 1st, 2009, http://aidsinfo.nih.gov
3. Clumeck N, Pozniak A, Raffi F, and the European Aids Clinical Society (EACS) Executive Committee, *Guidelines for the Clinical Management and Treatment of HIV Infected Adults in Europe 2009*, version 5.2 http://www.europeanaidsclinicalsociety.org/guidelinespdf/1_Treatment_of_HIV_Infected_Adults.pdf
4. BG Gazzard on behalf of the BHIVA Treatment Guidelines Writing Group*, British HIV Association guidelines for the treatment of HIV-1-infected adults with antiretroviral therapy 2008, HIV Medicine (2008), 9, 563–608
5. Linee Guida italiane sull'utilizzo degli antiretrovirali nelle persone con infezione HIV. http://www.salute.gov.it/hiv/newsHiv.jsp?id=1114&menu=inevidenza&lingua=italiano

**STUDY ASSESSMENT AND PROCEDURES**

**Flow-chart/Time and Events Schedule**

| **Procedures** | **Screening**  **(day -28)** | **Basale**  **(giorno 0)** | **Follow-up visit (weeks 4, 12, 36)** | **Follow-up visit (week**  **24)** | **Follow-up visit (week 48)** | **Follow-up visit**  **(week s**  **60, 72, 84)** | **Follow-up visit ( week 96)** | **Follow –up vist post-treatment**  **(after 30 days)** |
| --- | --- | --- | --- | --- | --- | --- | --- | --- |
| Written informed consent | **X** |  |  |  |  |  |  |  |
| Medical history | **X** |  |  |  |  |  |  |  |
| Therapeutic history | **X** |  |  |  |  |  |  |  |
| Concomitant medication use | **X** | **X** | **X** | **X** | **X** | **X** | **X** | **X** |
| HBsAg ed anti-HCV | **X** |  |  |  |  |  |  |  |
| Serum pregnancy test or serum FSH | **X** |  |  |  |  |  |  |  |
| Full physical examination, vital signs, height, weight | **X** | **X** | **X** | **X** | **X** | **X** | **X** | **X** |
| Blood exam and urine analysis | **X** | **X** | **X** | **X** | **X** | **X** | **X** | **X** |
| Urine analysis (24 hours) |  | **X** |  | **X** | **X** | **X****** | **X** |  |
| Hematology chemistry | **X** | **X** | **X** | **X** | **X** | **X** | **X** | **X** |
| Lymphocyte subsets and HIV RNA | **X** | **X** | **X** | **X** | **X** | **X** | **X** | **X** |
| Darunavir, ritonavir and maraviroc TDM (arm a)*** |  |  | **X** | **X** | **X** | **X** | **X** | **X** |
| Whole blood sample for viral DNA genotyping | **x** | **X** | **X** | **x** | **x** | **x** | **x** |  |
| Blood sample for HIV resistance test ant tropism test | **(*)** | **(*)** | **(*)** | **(*)** | **(*)** | **(*)** | **(*)** |  |
| Fasting insulin, study of bone metabolism |  | **X** |  | **X** | **X** | **X** | **X** |  |
| **IMT, FMD, body fat |  | **X** |  |  | **X** |  | **X** |  |
| **Bone DEXA |  | **X** |  |  | **X** |  | **X** |  |
| Adherence self-reported questionnaire |  | **X** | **X** | **X** | **X** | **X** | **X** |  |
| Symptoms questionnaire and QoL |  | **X** | **X (only week 4)** | **X** | **X** | **X** | **X** |  |
| **Neurocognitive test |  | **X** |  |  | **X** |  | **X** |  |

(*) sample storage at -20°C and for analysis in virological failure (two consecutive measures of HIV-RNA higher than 50 copies/mL or a single measure higher than 1000 copies/mL)

** in selected participating centers

*** TDM in 20 patients at week 4 with 9 points of determination **** at week 72
